# Extracellular vesicle‐encapsulated miR‐30c‐5p reduces aging‐related liver fibrosis

**DOI:** 10.1111/acel.14310

**Published:** 2024-09-13

**Authors:** Alice C. Rodrigues, Yujing J. Heng, Frank J. Slack

**Affiliations:** ^1^ Department of Medicine, Beth Israel Deaconess Medical Center Harvard Medical School Boston Massachusetts USA; ^2^ Department of Pharmacology Universidade de Sao Paulo Instituto de Ciencias Biomedicas São Paulo Brazil; ^3^ Department of Pathology, Beth Israel Deaconess Medical Center Harvard Medical School Boston Massachusetts USA; ^4^ Harvard Medical School Initiative for RNA Medicine Harvard Medical School Boston Massachusetts USA

**Keywords:** aging, exercise, extracellular vesicles, fibrosis, microRNAs, miR‐30

## Abstract

Aging is associated with decreased health span, and despite the recent advances made in understanding the mechanisms of aging, no antiaging drug has been approved for therapy. Therefore, strategies to promote a healthy life in aging are desirable. Previous work has shown that chronic treatment with extracellular vesicles (EVs) from young mice prolongs lifespan in old mice, but the mechanism of action of this effect on liver metabolism is not known. Here we investigated the role of treatment with EVs derived from young sedentary (EV‐C) or exercised (EV‐EX) mice in the metabolism of old mice and aimed to identify key youthful‐associated microRNA (miRNA) cargos that could promote healthy liver function. We found that aged mice treated with either EV‐C or EV‐EX had higher insulin sensitivity, higher locomotor activity resulting in longer distance traveled in the cage, and a lower respiratory exchange ratio compared to mice treated with EVs from aged mice (EV‐A). In the liver, treatment with young‐derived EVs reduced aging‐induced liver fibrosis. We identified miR‐30c in the EVs as a possible youth‐associated miRNA as its level was higher in circulating EVs of young mice. Treatment of aged mice with EVs transfected with miR‐30c mimic reduced stellate cell activation in the liver and reduced fibrosis compared to EV‐negative control by targeting Foxo3. Our results suggest that by delivering juvenile EVs to old mice, we can improve their liver health. Moreover, we identified miR‐30c as a candidate for antiaging liver therapy.

## Graphical abstract

Extracellular vesicles from young sedentary (EV‐C) or exercised (EV‐EX) individuals when delivered to aged subjects protect them from developing advanced liver fibrosis as observed with EV‐A by reducing the activation of HSCs. The mechanism of the fibrosis regression seems to differ between EV‐C and EV‐EX. While EV‐C potentially induces HSC senescence, EV‐EX reduces inflammation and accelerates elastin degradation. The hepatic levels of miR‐30c‐5p were correlated with HSC activation as shown by the protective effect of EV‐miR‐30c in liver fibrosis.

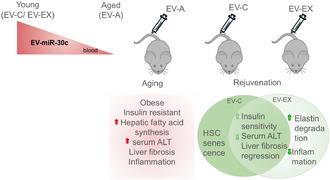

**Abbreviations**
Acaca: acetyl‐Coenzyme A carboxylase alpha
AKT: activates protein kinase B
ALT: alanine aminotransferases
AST: aspartate aminotransferases
AUC: Area under the curve
CD81: CD81 antigen
CD9: CD9 antigen
CEBPA/Cebpa: CCAAT enhancer binding protein alpha
Cs: citrate synthase
Dgat1: diacylglycerol O‐acyltransferase 1
EGFR: Epidermal growth factor receptor
EV: extracellular vesicles
EV‐A: extracellular vesicles from aged mice
EV‐C: extracellular vesicles from young mice
EV‐EX: extracellular vesicles from young and exercised mice
Fasn: fatty acid synthase
FC: Fold change
FDR: False discovery rate
FOXO: forkhead box, sub‐group O
*G6Pase*
: Glucose 6‐phosphatase
GAPDH: Glyceraldehyde‐3‐phosphate dehydrogenase
HSC: Hepatic Stellate Cell
IRS: insulin receptor substrate
ITT: Insulin Tolerance Test
Lipe: lipase, hormone sensitive
miR: microRNA
MMP: metalloproteinase
MRI: Magnetic resonance imaging
MTS: Masson's Trichrome staining
NAD: Nicotinamide adenine dinucleotide
NAFLD: Nonalcoholic fatty liver disease
NAMPT: Nicotinamide phosphoribosyltransferase
PBS: Phosphate‐buffered saline
*Pepck1*
: phosphoenolpyruvate carboxykinase 1
PPARG: Peroxisome proliferator‐activated receptor gamma
RER: respiratory exchange ratio
SASP: Senescence‐Associated Secretory Phenotype
Scd1: stearoyl‐Coenzyme A desaturase 1
Srebpf1: sterol regulatory element binding transcription factor 1
TG: triglyceride
TIMP: Metallopeptidase inhibitor
VEGF: Vascular endothelial growth factor
WAT: white adipose tissue

## 1 INTRODUCTION

Life expectancy at birth has been increasing progressively and so has the number of people aged 60 years or older. There will be 2.1 billion people who are 60 years or older by 2050 (Noto, 2023), and while people are living longer, they are not necessarily living healthier. The healthy life expectancy (HALE) at age 60 ranges from 9.8–20.4 years compared to 13.2–26.2 years for life expectancy. Therefore, strategies that can promote a healthy life in aging are desirable.

Current efforts to understand the molecular mechanism of aging and factors capable of delaying or accelerating aging include conducting parabiosis experiments, which is a surgical technique that unites the vasculature of two animals. Heterochronic parabiosis between young and old mice has shown that the young circulatory milieu is capable of reversing aging‐related conditions, suggesting that there are juvenile factors that could be used to prevent some aging‐related phenomena and extend lifespan (Zhang et al., 2023). Despite the encouraging results of parabiosis, so far, no soluble molecule has met the criteria for an antiaging factor. Additional research to identify circulating pro‐youth or pro‐aging factors is crucial to understand the molecular mechanisms of aging and to propose strategies to delay those mechanisms and extend the health span.

Extracellular vesicles (EVs) are present in circulation and can be biomarkers of aging (Kern et al., 2023) as their cargo largely depends on the functional state of cells producing them. Another key function of EVs is the intercellular and interorgan transfer of proteins and microRNAs (miRNAs) (Isaac et al., 2021). EVs containing nicotinamide phosphoribosyltransferase (eNAMPT) have been suggested as a potential antiaging intervention (Yoshida et al., 2019). EVs contain miRNAs (Chitti et al., 2024), and our group has made seminal contributions on miRNAs that regulate longevity in *C*. *elegans* (Boehm & Slack, 2005; Matai et al., 2023) and in humans (Smith‐Vikos et al., 2016). Exercise, a common lifestyle intervention to promote healthier aging, has been shown to increase the release of EVs in circulation (Chong et al., 2022). Furthermore, the composition of miRNAs in serum EVs is different between aged and young mice (Lee et al., 2018), and EVs containing miRNAs may be related to the rate of aging (Kern et al., 2023; Zhang et al., 2017). Recently, it has been shown that miRNAs in plasma EVs are a rejuvenation factor in blood of young mice and have the potential to decrease cell senescence, stimulate PPARG coactivator 1 alpha expression, improve mitochondrial energetic metabolism and mitigating mitochondrial deficits in aged tissues (Chen et al., 2024).

Particularly in the liver, hepatic cell senescence has been implicated in liver disease progression (Aravinthan & Alexander, 2016). Hepatocyte senescence can cause steatosis by inducing mitochondrial dysfunction resulting in reduced fat metabolism (Ogrodnik et al., 2017). Cellular senescence is a state of irreversible cell‐cycle arrest in response to stresses and the acquisition of a senescence‐associated secretory phenotype (SASP). SASP factors from senescent hepatocytes consist of an array of chemokines, cytokines, proteases and growth factors that can trigger hepatic stellate cell (HSC) activation and induce fibrogenesis (Wijayasiri et al., 2022). The transcription factor nuclear factor‐kappa B (NF‐kB) can drive SASP and also is a master regulator of hepatic injury and liver fibrosis as it can regulate survival and activation of HSCs (Luedde & Schwabe, 2011; Salminen et al., 2012).

Insulin resistance is another factor commonly seen in aged mice that is associated with mitochondrial dysfunction. In healthy liver tissue, insulin binding to the insulin receptor recruits insulin receptor substrate (IRS) 1/IRS2, which activates protein kinase B (AKT), stimulates fatty acid synthesis, and inhibits gluconeogenesis. In mouse insulin resistance, it has been shown that a hepatic selective insulin resistance is due to an imbalance of *Irs1* and *Irs2* expression; while *Irs2* is downregulated and insulin‐mediated suppression of gluconeogenesis is impaired, *Irs1* is overexpressed and an uncontrolled increase in hepatic lipogenesis persists (Kubota et al., 2017).

In this manuscript, we tested the hypothesis that aged mice treated with EVs from young mice would improve their liver health span. Additionally, we tested whether treating old mice with EVs from young, acutely exercised mice confer an extra benefit to their health? We found that EVs from young sedentary or acutely exercised mice, when delivered to aged mice, improve insulin sensitivity and distance traveled in the cage as well as delay aging‐induced liver fibrosis. We also demonstrated that circulating miR‐30c might be an antiaging factor as it was increased in the serum of young animals, and its reduced levels in aging liver was associated with the accelerated progression of liver fibrosis. Finally, treating aged mice with EVs from aged mice containing miR‐30c mimic decreased HSC activation and mitigated liver fibrosis in aged mice.

## 2 RESULTS

### 2.1 Aged mice are obese and insulin‐resistant

Aging increases the risk for obesity and type 2 diabetes, and at the same time, obesity and type 2 diabetes are causes of accelerated aging in humans. We compared the body weight and composition of two‐month‐old (young) and 18‐month‐old (aged) mice and their response to insulin (Figure S1). Aged mice developed spontaneous obesity and insulin resistance compared to young mice as evidenced by higher body weight due to increased adiposity (Figure S1A,B, all *p* < 0.05). Fasting glucose levels were lower in aged mice probably due to an impairment in gluconeogenesis as *Pepck1* and *G6Pase* transcripts levels were reduced in the liver of aged mice compared to the young mice (Figure S1C,F, all *p* < 0.05). Aged mice had higher glucose levels after insulin injection as measured by an insulin tolerance test (ITT) and as a result higher area under the curve (AUC) of ITT (Figure S1D,E, all *p* < 0.05). Because glucose disappearance in the circulation of aged mice is lower, these data suggest that aged mice have less insulin sensitivity.

### 2.2 Treatment of aged mice with EVs from young mice improved their metabolism and locomotor activity

EVs obtained from the pooled serum of young sedentary (EV‐C), young acutely exercised (EV‐EX), and aged mice (EV‐A) presented with an average size of 89 ± 14 for EV‐C, 86 ± 46 nm for EV‐EX, and 104 ± 71 nm for EV‐A (Figure S1G). Their concentrations in particles/mL were EV‐C: 2.50 × 10^11^ ± 3.44 × 10^10^, EV‐EX: 4.48 × 10^11^ ± 3.08 × 10^9^, and EV‐A: 1.79 × 10^11^ ± 2.86 × 10^10^ (Figure S1G). We measured some markers and possible contaminants to characterize EVs according to the International Society of Extracellular Vesicles guidelines (Figure S1H–M) (Lucien et al., 2023). EVs were positive to the EV markers Alix and CD81 but not CD9 (Figure S1H–J). To evaluate the purity of the EVs preparation, we measured albumin, apolipoprotein A1 (ApoA‐1), and ApoB (B‐48 and B‐100), contaminant proteins frequently co‐isolated with EVs. Neither albumin nor Apoa1 was detected in the EV suspension (Figure S1K,L). However, ApoB‐48 (265 kDa) and ApoB‐100 (550 kDa) were found in our preparation, and ApoB‐48 was more abundant in EV samples compared to total serum while ApoB‐100 was most abundant in EV‐C, followed by total serum, and minimally detected in EV‐EX and EV‐A (Figure S1K). Considering that albumin is the most abundant protein in the circulation, our preparations were pure; however, the presence of ApoB is something to consider as they are carriers of miRNAs.

**FIGURE 1:**
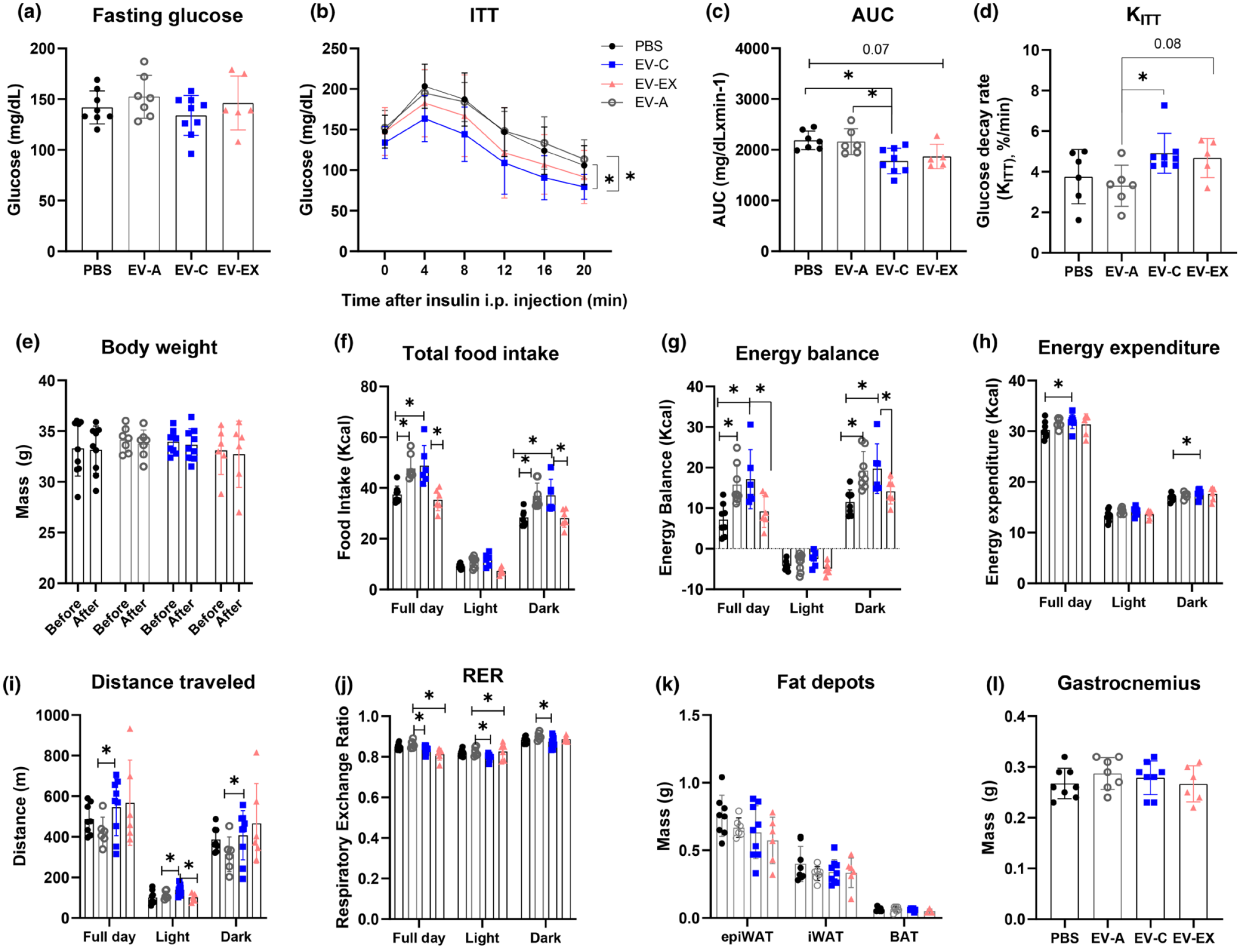
Treatment of aged mice with extracellular vesicles (EVs) from young mice improved their metabolism and distance traveled. Eighteen‐month‐old male mice were intravenously injected PBS or EVs from aged (EV‐A), young sedentary (EV‐C) or young exercised (EV‐EX) mice weekly for 4 weeks. (a) Fasting glucose levels. (b) Insulin tolerance test (ITT) was performed after 5 h of fasting in the last week of treatment. (c, d) Area under the curve (AUC) and insulin tolerance rate decay (K_ITT_) calculated from the ITT curve in (b). (e) Body weight before and after EV treatment. (f–j) Data obtained from indirect calorimetry in the dark/light cycles and total period performed after the last injection. Food consumption (f); energy balance (g); energy expenditure (h); distance traveled in cage (i) and respiratory exchange ratio (RER) (j). (k,l) Fat depots and gastrocnemius muscle collect after euthanasia and weighed. Three experimental cohorts were performed with two or three animals in each group. Each symbol inside the column represents one animal *n* = 7–8 (PBS), *n* = *6*–7 (EV‐A), *n* = 8–9 (EV‐C) and *n* = 5–6 (EV‐EX). *indicates *p* < 0.05 after performing Tukey's post hoc test (a–e; i–j), ANCOVA (F‐H).

After EV characterization and quantification, the same amount (10^9^ particles) of EV‐A, EV‐C or EV‐EX, or PBS (vehicle) was used to intravenously treat randomly picked 18‐month‐old mice once a week for 4 weeks. This concentration was chosen based on a previous study (Castaño et al., 2020). There was no difference in fasting or ITT blood glucose levels among aged mice treated with PBS, EV‐A, EV‐C, or EV‐EX (Figure 1a, *p* = 0.3662 and Figure 1b, *p* = 0.5252, respectively). However, aged mice treated with EV‐C displayed higher insulin sensitivity as observed by lower AUC (Figure 1c; EV‐C *p* = 0.02) and a higher plasma glucose disappearance rate (K_ITT_) calculated from ITT curve when compared to EV‐A and PBS groups (Figure 1d; *p* = 0.02 and *p* = 0.01, respectively). Aged mice treated with EV‐EX had lower AUC and higher K_ITT_ compared to PBS and EV‐A but were not statistically significantly different (Figure 1c,d; *p* = 0.07 and *p* = 0.08).

Higher insulin sensitivity was not due to differences in body weight (Figure 1e; *p* = 0.9975). Despite no difference in body mass, compared to PBS and EV‐EX‐treated mice, aged mice that received EV‐C or EV‐A had higher food intake, leading to a more positive energy balance (Figure 1f,g, all *p* < 0.05). Energy expenditure was also higher in aged mice that received EV‐C compared to PBS (Figure 1h, all *p* < 0.05). When compared to EV‐A‐treated mice, treatment with EVs from young mice induced longer distance traveled in the cage and lower RER (Figure 1i *p* = 0.0274 and Figure 1j, all *p* < 0.05, respectively). Although there was a difference in the RER, fat depot mass or gastrocnemius mass did not change among the groups treated with PBS and EVs (Figure 1k,l, both *p* > 0.05).

**FIGURE 2:**
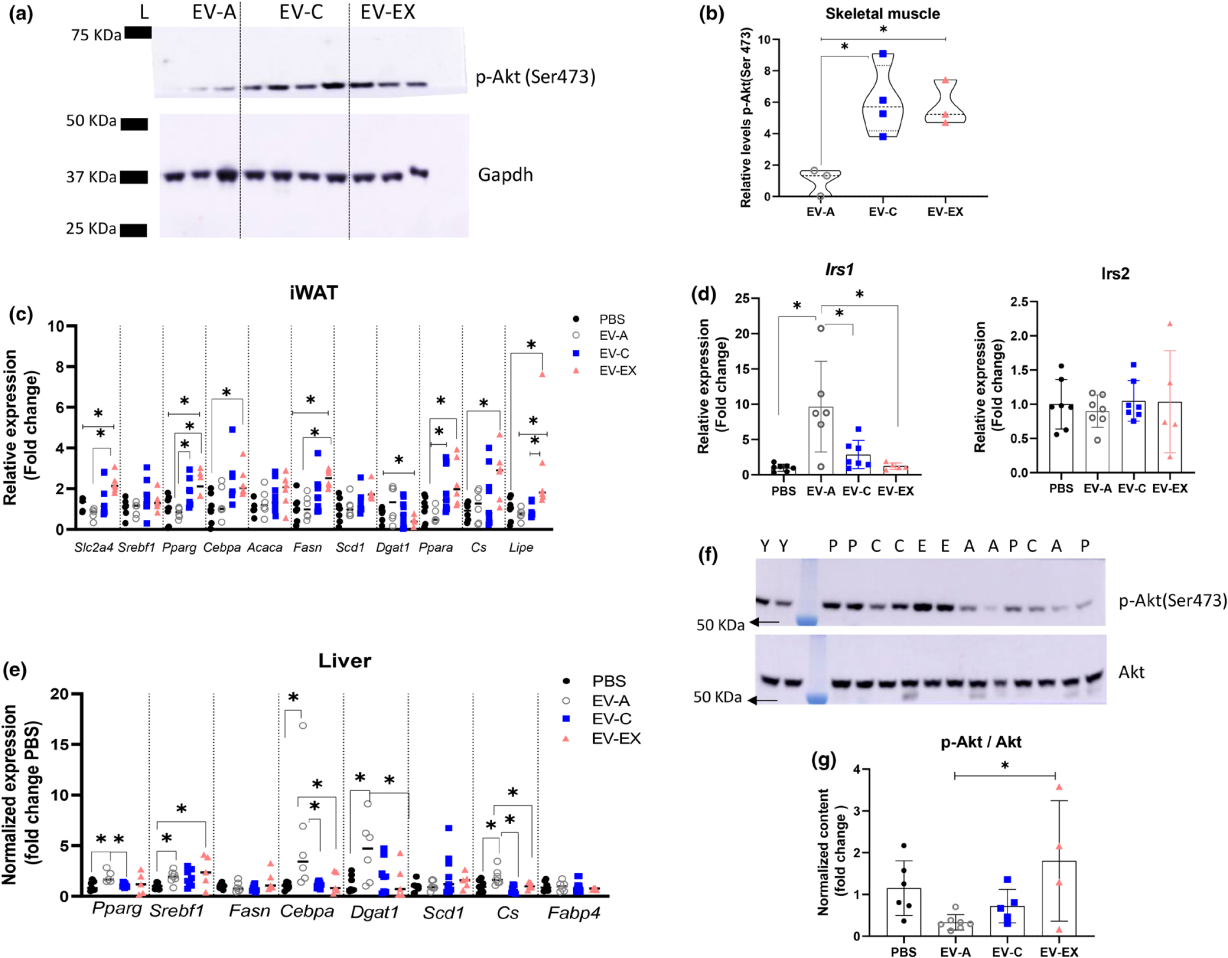
Effect of the treatment of aged mice with extracellular vesicles from young (EV‐C), young exercised (EV‐EX), and aged mice (EV‐A) in key insulin‐sensitive tissues‐ skeletal muscle, liver, and adipose tissue. (a) Western blot image of phospho‐Akt (Ser473) (p‐Akt (Ser 473)) and Gapdh in the skeletal muscle of EV‐A (*n* = 3), EV‐C (*n* = 4) and EV‐EX (*n* = 3) groups. PBS samples were not detected and excluded from this panel. (b) Quantification of p‐Akt was performed after normalization with Gapdh. Relative levels to the EV‐A group are shown. (c) Transcript levels of genes related to fatty acid synthesis and oxidation in the inguinal adipose tissue (iWAT). Each symbol inside the column represents one animal *n* = 4–7 (PBS), *n* = 5–7 (EV‐A), *n* = 6–8 (EV‐C), and *n* = 5–6 (EV‐EX). (d, e) Transcript levels of *Irs1* and *Irs2* (d) and genes related to fatty acid metabolism (e) measured by RT‐qPCR in the liver of aged mice treated with EVs. (f) Representative image of the western blot of p‐Akt (Ser473) and Akt from liver protein of young (y), aged treated with PBS (P), aged treated with EV‐C (C); aged treated with EV‐EX (E). (g) Quantification of phospho‐Akt/Akt ratio of all samples in the liver. From (d–g) Each symbol inside the column represents one animal *n* = 6–7 (PBS), *n* = 5–6 (EV‐A), *n* = 5–7 (EV‐C) and *n* = 4–6 (EV‐EX). *indicates *p* < 0.05 after performing Tukey's post hoc test.

We evaluated gene expression and/or phospho‐Akt(ser473) (p‐Akt) in the skeletal muscle, inguinal white adipose tissue (iWAT), and liver to gain insight into the mechanisms for improved insulin sensitivity. In the skeletal muscle of aged mice, we observed that EV‐C and EV‐EX treatment increased p‐Akt levels compared to those that received EV‐A suggesting improved insulin sensitivity (Figure 2a,b all *p* < 0.05). In the iWAT, we evaluated some transcripts of genes related to glucose uptake (*Slc2a4‐*glucose transporter type 4) and lipid metabolism (*Pparg* – peroxisome proliferator‐activated receptor gamma; *Cebpa* – CCAAT enhancer binding protein alpha; *Srebpf1* – sterol regulatory element binding transcription factor 1; *Acaca –* acetyl‐Coenzyme A carboxylase alpha; *Fasn‐* fatty acid synthase; *Scd1 –* stearoyl‐Coenzyme A desaturase 1; *Dgat1 –* diacylglycerol O‐acyltransferase 1; *Ppara* – Ppar alpha; *Cs – citrate synthase*, and *Lip –* lipase, hormone sensitive). EV‐A and EV‐C treatment did not modulate the mRNA expression of those genes compared to PBS. Compared to EV‐A, EV‐C treatment induced *Pparg* and *Ppara* transcript levels (Figure 2c, all *p* < 0.05). EV‐EX treatment had unique effects on glucose and fatty acid metabolism‐related gene expression. Compared to PBS, treatment with EV‐EX induced the expression of *Slc2a4*, *Pparg*, *Cebpa*, *Fasn*, *Cs*, and *Lipe* (Figure 2c, all *p* < 0.05). In addition, it decreased the expression of *Dgat1* (Figure 2c, *p* < 0.05). Compared to EV‐A, EV‐EX induced *Slc2a4*, *Pparg*, *Fasn*, and *Lipe* mRNA expression (Figure 2c, all *p* < 0.05). These data suggest treatment with EV‐EX increased glucose uptake, mitochondrial activity, and lipolysis in the aged mice, all effects induced by exercise in the iWAT (Stroh & Stanford, 2023).

**FIGURE 3:**
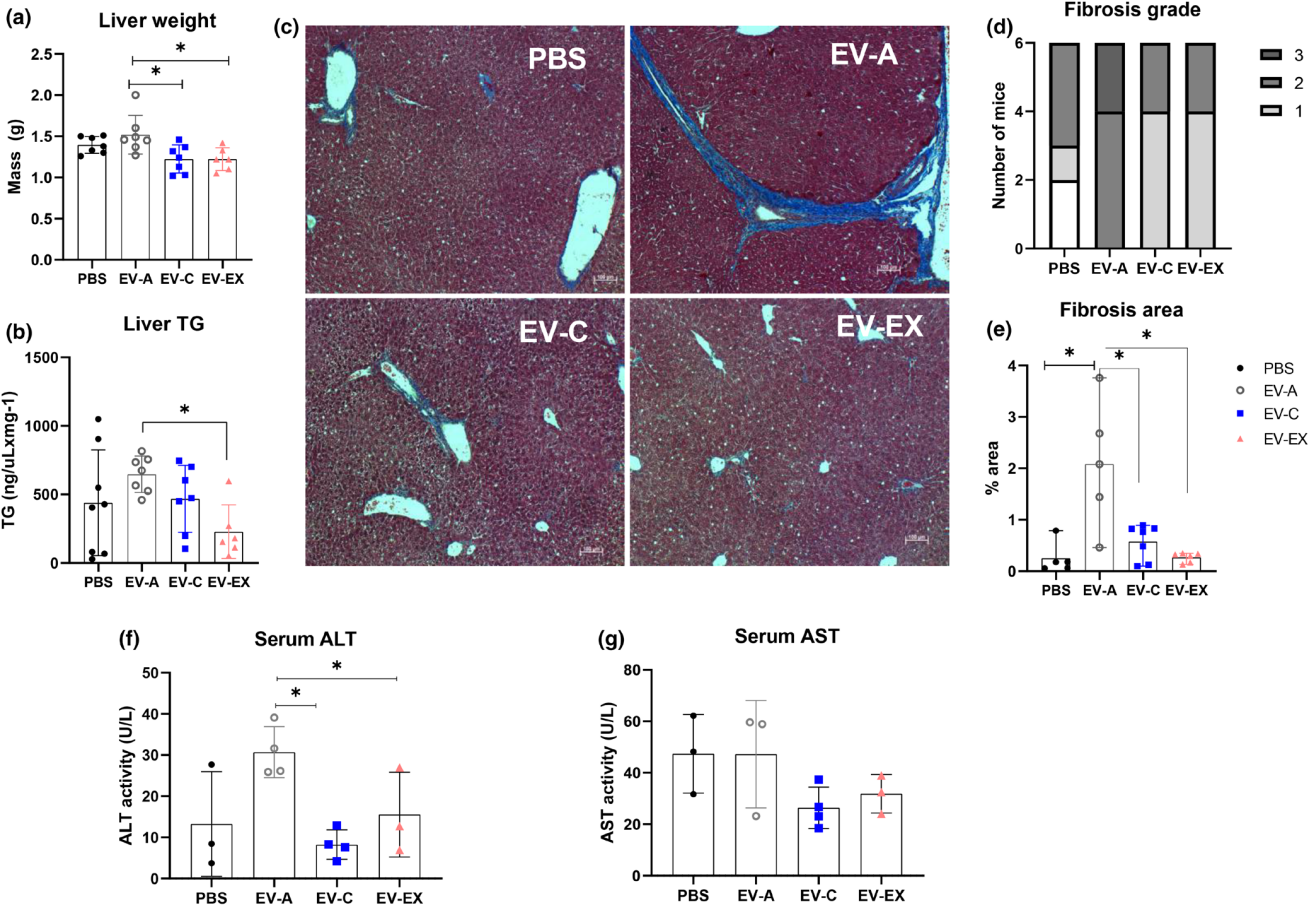
Treatment of aged mice with EV from young mice improves aging‐induced liver fibrosis. Eighteen‐month‐old male mice were intravenously injected PBS or extracellular vesicles from aged (EV‐A), young sedentary (EV‐C) or young exercised (EV‐EX) mice weekly for 4 weeks. (a, b) Liver weight and liver triglyceride (TG) content. (c) Representative photomicrographs of liver stained with Masson's Trichrome from aged mice treated with PBS or EVs. (d) Calculated fibrosis grade from each animal, from 0 to 4. No animals were scored 4. (e) Fibrosis area calculated using ImageJ. (f, g) Serum alanine transaminase (ALT) (f) and aspartate transaminase (AST) (g) enzyme activities. Serum with hemolysis were excluded as interferes with the measurement. Each symbol inside the column represents one animal *n* = 3–8 (PBS), *n* = 4–7 (EV‐A), *n* = 3–8 (EV‐C) and *n* = 3–6 (EV‐EX). *p < 0.05 as indicated by Tukey's post hoc test.

We also investigated the effect of treatment with EVs in the liver (Figure 2d–g). Compatible with a great increase in *Irs1* mRNA levels induced by EV‐A treatment compared to all the other groups (Figure 2d), *Pparg*, *Srebp1c*, *Cebpa*, *Dgat1*, and *Cs* were induced in the liver of aged mice treated with EV‐A, suggesting that fatty acid synthesis was induced (Figure 2e, all *p* < 0.05). Compared to EV‐A, EV‐C treatment reduced *Irs1*, *Pparg*, *Cebpa* and *Cs* expression and EV‐EX treatment reduced *Irs1*, *Cebpa*, *Dgat1* and *Cs* (Figure 2d,e, all *p* < 0.05). None of the treatments modulated the transcription of *Fasn*, *Scd1*, *Fabp4*, and *Irs2* (Figure 2d,e; *p* > 0.05). Only treatment with EV‐EX increased p‐Akt levels compared to EV‐A, suggesting EV‐EX‐treated liver had improved insulin sensitivity (Figure 2f,g; *p* = 0.02). Liver weight in aged mice that received young‐derived EVs was significantly reduced compared to EV‐A (Figure 3a, *p* < 0.05). We measured liver triglyceride (TG) levels (Figure 3b). Aged mice that received EV‐EX had significantly reduced TG concentration in their liver (Figure 3b, *p* = 0.03), consistent with the decrease in *Dgat1*, a key enzyme in TG synthesis.

Taken together, aged mice treated with EV‐A have reduced insulin sensitivity, reduced locomotion, and increased fatty acid synthesis towards accumulation. Their livers displayed altered lipid metabolism indicative of accelerated cell senescence since senescent cells present higher metabolic activity towards fatty synthesis (Ogrodnik et al., 2017). On the other hand, when treated with young EVs, aged mice have higher insulin sensitivity, are more active, and fatty acid synthesis is attenuated. Particularly, EV‐EX conferred an extra benefit as it improved insulin sensitivity in all three main insulin‐sensitive tissues. Therefore, treatment with EVs from old or young mice seems to recapitulate the features of an old and young subject. In addition, treatment with EVs derived from aerobically treated mice mimics some effects of exercise in the liver, skeletal muscle, and iWAT.

### 2.3 Treatment of aged mice with EVs from young mice improves liver fibrosis by activating fibrosis resolution mechanisms

Liver sections stained with Hematoxylin and Eosin (H&E) revealed that steatosis was not predominant in aged mice treated with PBS and did not differ among the groups treated with EVs (data not shown). However, areas of significant fibrosis were observed. Masson's Trichrome staining (MTS) confirmed that 66.6% of PBS‐treated livers had aging‐induced fibrosis. Treatment with EV‐A accelerated the degree of fibrosis (Figure 3c–e). Treatment of aged mice with young EVs (EV‐C and EV‐EX) did not increase the fibrotic area or fibrosis grade compared to PBS, but fibrosis was reduced when compared to those that received EV‐A (Figure 3d, *χ* ^2^ = 18.67, 9; *p* = 0.02 and Figure 3e, all *p* < 0.01).

We measured alanine and aspartate aminotransferases (ALT and AST, respectively) activity in the serum as markers of liver damage (Figure 3f,g). EV‐A had increased ALT activity compared to PBS (Figure 3f, *p* = 0.05) and EV‐C and EV‐EX groups (Figure 3f, *p* = 0.003 and *p* = 0.04, respectively). AST was not statistically different among the groups (Figure 3g).

**FIGURE 4:**
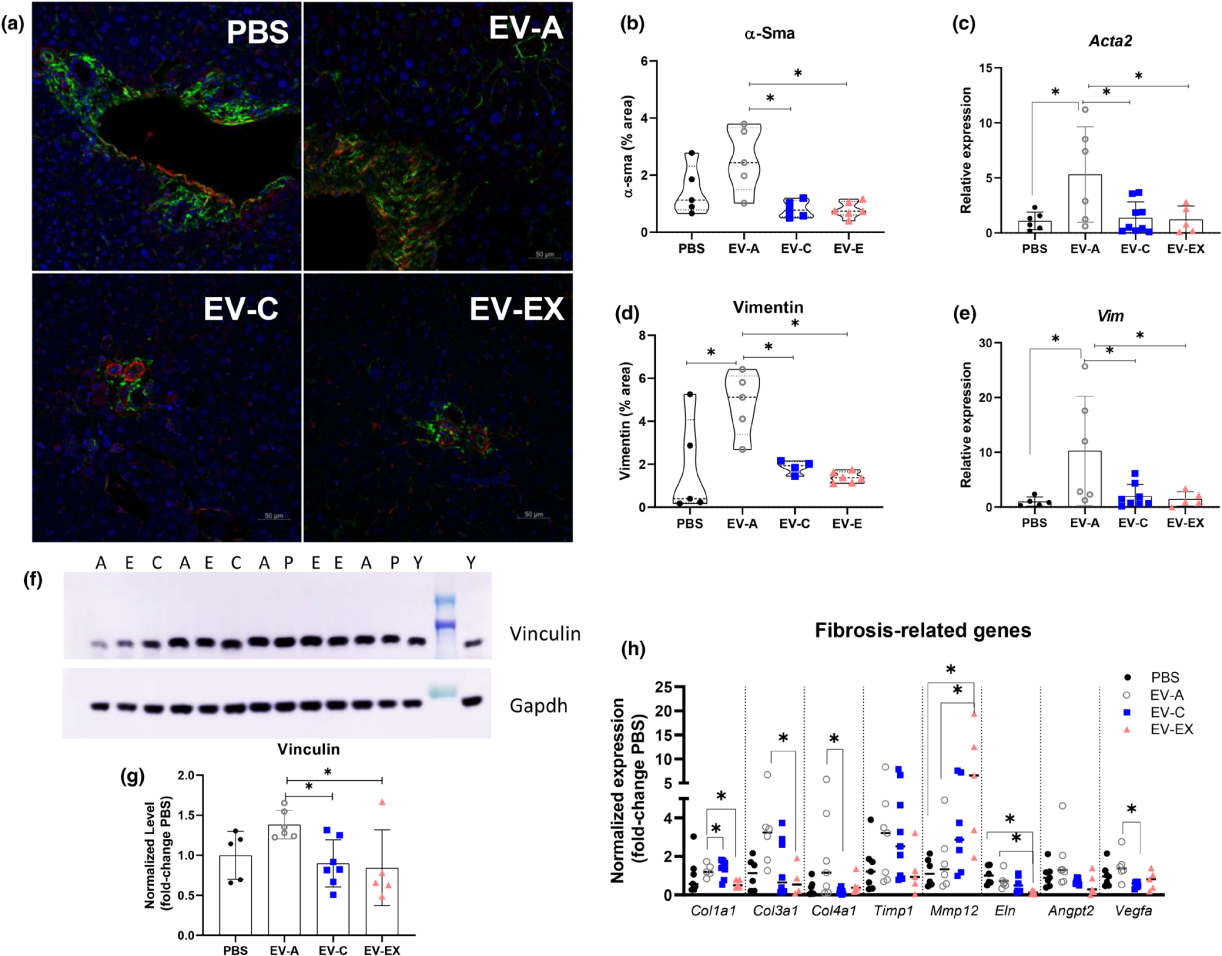
Treatment of aged mice with EV from young mice reduces hepatic stellate cells activation. (a) Livers from aged mice treated with PBS or extracellular vesicles from aged (EV‐A), young sedentary (EV‐C), or young exercised (EV‐EX) were harvested and fixed in 10% formalin for 48 h and subjected to immunofluorescence (IF) staining of alpha‐smooth muscle Actin (α‐Sma) in red and vimentin in green. Hoechst in blue stained the nucleus. (b, d) If quantification of the percentage of the area stained was performed using ImageJ software. (c, e) Transcript levels of *Acta2* and Vimentin (Vim) in the liver of treated mice measured by RT‐qPCR. (f) Representative bands for vinculin and Gapdh from protein of young (y), aged treated with PBS (P), aged treated with EV‐C (C); aged treated with EV‐EX (E) by western blotting. (g) Quantification of vinculin. Vinculin content was normalized to Gapdh content and was expressed relative to the PBS group. (h) Transcript levels of genes related to fibrosis measured by RT‐qPCR in the liver of aged mice treated with PBS or EVs. Each symbol inside the column represents one animal *n* = 5–8 (PBS), *n* = *5*–7 (EV‐A), *n* = 5–8 (EV‐C), and *n* = 5–6 (EV‐EX). *p < 0.05 as indicated by Tukey's post hoc test.

We further confirmed the higher degree of fibrosis in mice treated with EV‐A compared to EV‐C and EV‐EX by evaluating α‐Sma (*Acta2* gene), a marker of activated HSCs (Figure 4a and Figure 4b, *p* = 0.072 and *p* = 0.045, respectively). Accordingly, transcription levels of *Acta2* were increased in the liver of the EV‐A group compared to PBS, EV‐C, and EV‐EX (Figure 4c, all *p* < 0.05).

Vimentin, a marker of epithelial‐mesenchymal transition, was significantly increased in the livers of EV‐A‐treated mice compared to PBS, EV‐C, and EV‐EX at protein (Figure 4a,d, all *p* < 0.05) and transcription levels (Figure 4e, all *p* < 0.05). Vinculin, a focal adhesion marker, was also increased in EV‐A‐treated mice compared to mice that received EV‐C or EV‐EX (Figure 4f and Figure 4g, *p* = 0.04 and *p* = 0.03, respectively).

In light of the histological findings, we quantified the mRNA levels of fibrosis‐related genes. In general, there was no differential expression of fibrotic genes between PBS and EV‐A; however, treatment with EV‐C and EV‐EX reduced the expression of collagen type 1 alpha 1 (*Col1a1*) (Figure 4h, all *p* < 0.05). Compared to EV‐A, EV‐C treatment reduced the expression of *Col4a1* and vascular endothelial growth factor A (*Vegfa*). Specifically, EV‐EX reduced *Col3a1* levels and increased the expression of metalloproteinase 12 (*Mmp12*) compared to EV‐A group (Figure 4h, all *p* < 0.05). Accordingly, elastin (*Eln*), a substrate for Mmp‐12, was downregulated by EV‐Ex treatment compared to PBS and EV‐A groups (Figure 4h, all *p* < 0.05). Metallopeptidase inhibitor 1 (*Timp1*) and angiopoietin‐2 (*Angpt2*) expression were not different among the groups (Figure 4h, *p* > 0.05).

During fibrosis regression, Mmp‐12, also called macrophage elastase, is predominantly expressed in macrophages that acquire a restorative phenotype characterized by low Ly‐6C expression (Ramachandran et al., 2012). Mmp‐12 has been associated with decreased activation of HSC and decreased pro‐inflammatory cytokines (Wang et al., 2020).

**FIGURE 5:**
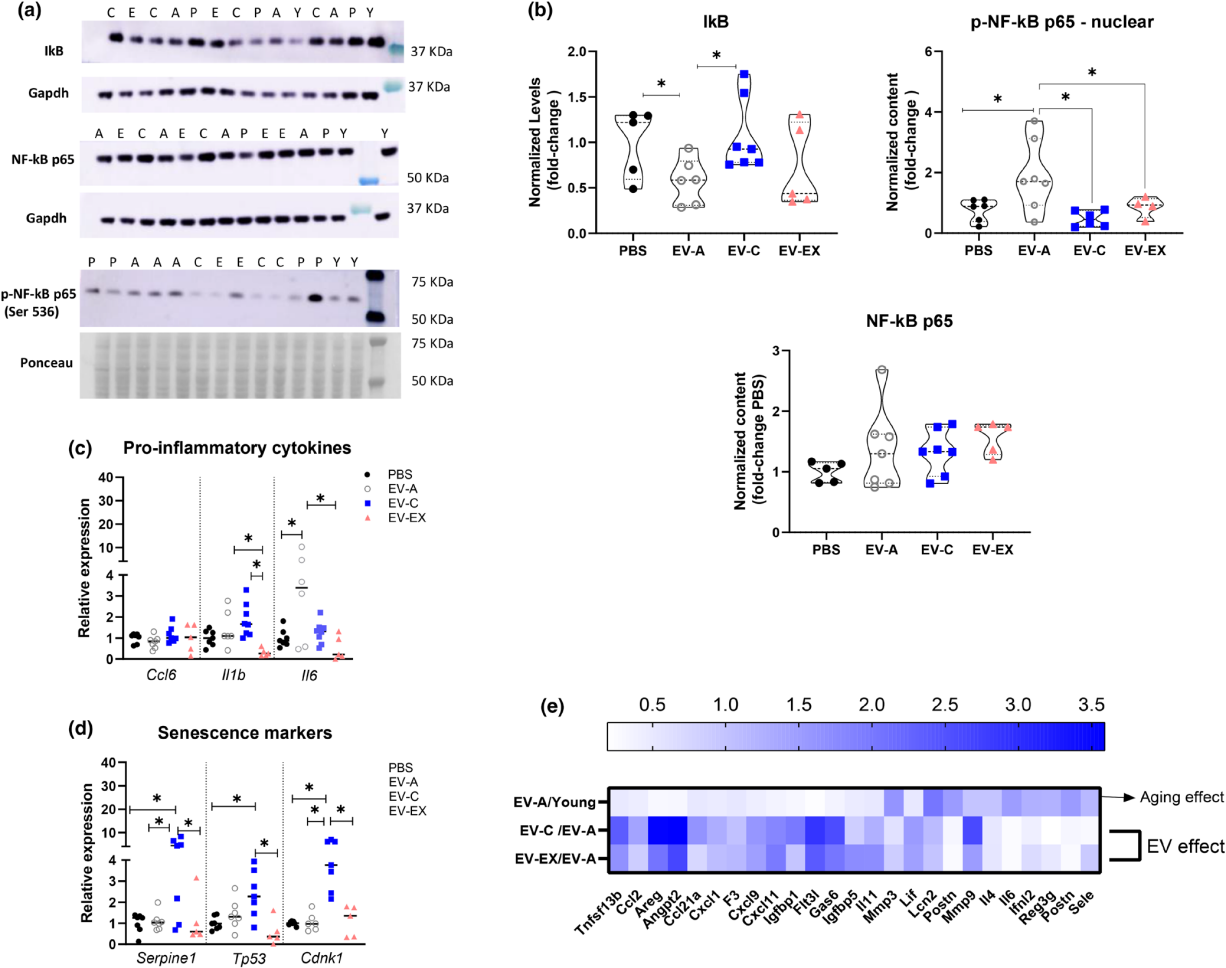
Treatment of aged mice with EV from young mice promotes fibrosis resolution. Livers from aged mice treated with PBS or extracellular vesicles (EVs) from aged (EV‐A), young sedentary (EV‐C) or young exercised (EV‐EX) were harvested and used for protein or RNA extraction. (a) Representative bands of western blot for inhibitor of NF‐kB (IkB), NF‐kB p65, phospho‐NF‐kB p65 (Ser536), and Gapdh or total ponceau used as references from liver protein of young (Y), aged treated with PBS (P), aged treated with EV‐C (C); aged treated with EV‐EX (E). Gapdh gel bands in A represented under NF‐kB p65 bands were also used in Figure 4f, as the same membrane was used for the detection of both proteins of interest. (b) Quantification of the IkB, NF‐kB p65, and phospho‐NF‐kB p65 blots for all samples (*n* = 5/group). *p < 0.05 as indicated by Tukey's post hoc test. (c, d) Transcript levels of senescence‐associated secretory phenotype‐related genes in the liver measured by RT‐qPCR; (c) Pro‐inflammatory‐related genes; (d) Cellular senescence markers. Each symbol inside the column represents one animal *n* = 6 (PBS), *n* = 7 (EV‐A), *n* = 7 (EV‐C) and *n* = 5 (EV‐EX). *p < 0.05 as indicated by Tukey's post hoc test. (e) Profile of mean spot pixel density obtained from cytokine array immunoassay from young and aged mouse livers treated with EV‐A, EV‐C or EV‐EX. Ratio to 2‐m.o.‐mouse or to aged treated with EV‐A are shown in the heatmap (*n* = 1).

Therefore, we first measured the nuclear translocation of phospho‐NF‐kB p65 (p‐NF‐kB) as activation of NF‐kB is related to activation of inflammatory signaling pathways. Aged mice treated with EV‐A had lower protein expression of inhibitor of NF‐kB (IkB) and higher p‐NF‐kB in the nucleus compared to PBS, EV‐C, and EV‐EX groups (Figure 5a,b, all *p* < 0.05).

Then we measured the transcript levels of pro‐inflammatory cytokines and chemokines including interleukin 6 (*Il6*) and *Il1b*, and chemokine (C–C motif) ligand 6 (*CCl6*) (Figure 5c). When compared to the PBS‐treated old mice, EV‐A‐treated mice had significantly increased *Il6* expression compared to the PBS group; however, *CCl6 and Il1b* were not differentially expressed (Figure 5c), and compared to the EV‐A group, old mice that received EV‐EX had reduced expression of *Il1b* and *Il6* (Figure 5c, all *p* < 0.05). Compared to EV‐C, EV‐EX‐treated mice had lower expression of *Il1b* (Figure 5c, all *p* < 0.05).

Another mechanism of fibrosis resolution is the elimination of myofibroblasts. Activated HSCs can be deactivated, induced to apoptosis or senescence. Senescent‐activated HSCs remain metabolically active, exhibit gene expression profile consistent with the cell‐cycle exit, and secrete a variety of biologically active factors and cytokines that can regulate fibrosis and fibrosis resolution (Kisseleva & Brenner, 2020).

We measured senescence markers in the liver including p53 (*Trp53*), p21 (*Cdkn1a*), and plasminogen activator inhibitor 1 (*Serpine1*). Treatment of aged mice with EV‐A did not affect the transcription of those genes compared to the PBS‐treated group while those that received EV‐C had increased levels of *Serpine1*, *Tp53*, and *Cdkn1a* (Figure 5d, all *p* > 0.05). Compared to EV‐A, EV‐C treatment induced the expression of *Serpine 1* and *Cdkn1* expression and compared to EV‐EX, *Tpr53* and *Cdnk1* levels were higher (Figure 5d, all *p* < 0.05).

Then we chose one representative liver sample from each group to profile 111 mouse growth factors, cytokines, and chemokines using a membrane‐based antibody array (Figure 5e). Proteins with a ratio = |0.5| were related to fibrosis resolution. In general, proteins upregulated in the liver of aged mice treated with EV‐A were reduced by EV‐C or EV‐EX treatment.

Taken together, while both EV‐C and EV‐EX can induce liver fibrosis regression, they might trigger different mechanisms.

### 2.4 Serum EV‐miRNA content differs between aged and young mice

To gain a better insight into the health benefits of treating old mice with EVs from young animals, we profiled 109 mouse miRNAs of EV‐C, EV‐EX, and EV‐A (Figure S2A, Tables S2 and S3). No miRNAs were differentially expressed between any of the two groups at FDR < 0.05. At *p* < 0.05, six miRNAs were downregulated and three miRNAs were upregulated in EV‐EX when compared to EV‐A (Figure S2B). MiR‐375‐3p was downregulated in EV‐EX when compared to EV‐C (Figure S2C, *p* < 0.05). There were no miRNAs differentially expressed between EV‐C and EV‐A at *p* < 0.05. However, using Log2‐transformed fold‐change criteria of |2.0| revealed 13 miRNAs that exhibit substantial changes in their expression in EV‐C that could be biologically relevant despite the lack of statistical significance (Figure S2D).

**FIGURE 6:**
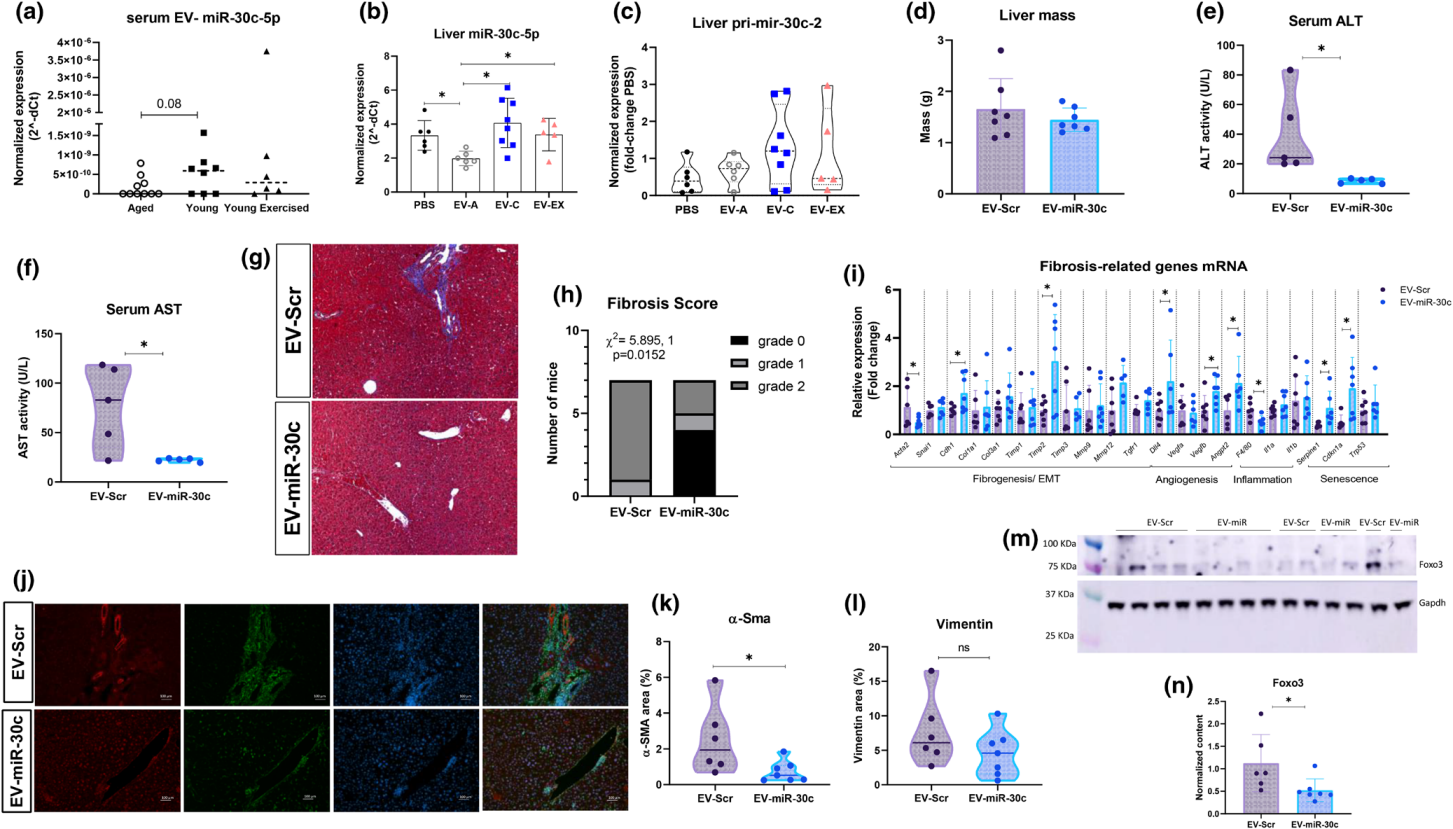
MiRNA‐30c is reduced in liver of aged mice with accelerated fibrosis and treatment with extracellular vesicle‐miR‐30c‐5p (EV‐miR‐30c) reduces liver fibrosis. Serum EVs were isolated from young (2 m.o.) sedentary (EV‐C) or acutely exercised (EV‐EX) and aged mice (18 m.o.) and total RNA was extracted for miR‐30c‐5p quantification (a) or used to treat aged mice. Each symbol inside the column represents one animal *n* = 11 (Aged), *n* = 8 (Young), *n* = 6 (Young exercised). (b, c) Mir‐30c‐5p mature and pri‐mir‐30c‐2 expression of aged mice treated with PBS or EVs (EV‐A, EV‐C or EV‐EX) measure by RT‐qPCR. Each symbol inside the column represents one animal *n* = 6 (PBS), *n* = 7 (EV‐A), *n* = 8 (EV‐C) and *n* = 5 (EV‐EX). (d–n) Data from aged mice treated with EV‐miR‐30c or EV containing a negative control (EV‐Scr). Each symbol represents one mouse (*n* = 7/group). (d) Liver mass after treatment. (e, f) Serum alanine transaminase (ALT) (e) and aspartate transaminase (AST) (f) enzyme activities. Two serums with hemolysis were excluded as erythrocytes have high ALT and AST activities and interfere with the measurement. (g) Representative photomicrographs of liver stained with Mason's Trichrome (MTS) from aged mice treated with EV‐Scr or EV‐miR‐30c. (h) Fibrosis score calculated from MTS images for each mouse; (i) Expression of mRNA of fibrosis‐related genes measured by RT‐qPCR. Levels were normalized with *Hprt1* and are expressed as fold‐change. The PBS group was used as a calibrator. (j) Representative photomicrographs of immunofluorescence for Vimentin (green) and α‐smooth Actin (α‐Sma) in red. Hoescht was used for nuclear staining (blue). (k, l) Quantification of α‐Sma and Vimentin performed using Image J software for each mouse. (m) Representative bands of western blot for Foxo3 and Gapdh. (n) Quantification of the optical density of the bands obtained in (m) using Image Studio. Levels of Foxo3 were normalized with Gapdh. EV‐mir: EV‐miR‐30c‐treated mice and EV‐Scr: EV‐Scrambled‐treated mice. *p < 0.05 as indicated by *t* test (d–n) or Tukey's post hoc test (a–b).

We examined the potential pathways associated with the miRNAs with a differential FC of |2.0|. We used IPA for pathway predictions from a set composed of 25 miRNAs upregulated and 22 miRNAs downregulated (Table S3). Hepatic System Disease was one of the most significant diseases associated with five of the miRNAs (Figure S2E), and particularly liver fibrosis was associated with miR‐30c‐5p. Next, we predicted miR‐30c‐5p mRNA targets and performed a core analysis to detect which pathways they are involved in. Pulmonary fibrosis, collagen biosynthesis, extracellular matrix organization, wound healing signaling, and hepatic fibrosis/HSC activation were the top five most significant pathways that included miR‐30c‐5p mRNA targets (Figure S2F and Table S4). We chose miR‐30c‐5p to conduct further investigations and validated its expression levels as increased in the EVs from young and young exercised (Figure 6a, *p* = 0.08). Other members of the miR‐30 family did not appear to be differentially expressed among the EV groups (Figure S2G).

### 2.5 miR‐30c‐5p is downregulated in the liver of aged mice treated with EV‐A

To relate changes in the liver with EV cargo content, we analyzed miR‐30c‐5p in the liver of aged mice that received PBS or EVs. Compared to PBS treatment, livers of old mice treated with EV‐A had lower levels of miR‐30c‐5p, an effect not observed in the liver of those treated with EV‐C or EV‐EX (Figure 6b, *p* = 0.01). When compared to the EV‐A group, miR‐30c‐5p levels are higher in mouse liver treated with EV‐C (*p* = 0.004) or EV‐EX (*p* = 0.03; Figure 6b). Changes in miR‐30c‐5p levels were not transcriptionally regulated as indicated by similar expression of *pri‐mir‐30c‐2* among the groups (Figure 6c, *p* = 0.22). *Pri‐mir‐30c‐1* expression was not detected in the liver (data not shown). Other members of the miR‐30 family did not appear to be regulated in the liver by the treatment with EVs (Figure S2H, all *p* > 0.05).

Vimentin, one of the validated targets of miR‐30c‐5p (Suh et al., 2014), was previously shown in Figure 3c to be upregulated in EV‐A group compared to PBS and, accordingly, reduced in EV‐C and EV‐E compared to EV‐A group.

### 2.6 EV‐miR‐30c‐5p treatment of aged mice reduced hepatic stellate cell activation

Because miR‐30c‐5p levels were low in the livers of aged mice that received EV‐A and were associated with a more advanced liver fibrosis than aged mice that received EV from young, we postulated that by delivering EV‐miR‐30c‐5p to the liver of aged mice, we could reverse aging‐induced liver fibrosis. Therefore, we modified EVs from aged mice to contain miR‐30c‐5p mimic (EV‐miR‐30c) or a scrambled sequence as a negative control (EV‐Scr) and treated old mice intravenously once a week for 4 weeks with 2.9 μg of miR‐30c‐5p mimic or scramble transfected into 10^9^ EV particles. We postulated that EV‐miR‐30c would mimic the effects of EV‐C. Treatment of aged mice with EV‐miR‐30c‐5p did not affect body weight and body composition or body weight gain (Figure S3A–C, all *p* > 0.05). No change in fasting glucose concentration was observed and, after challenge with insulin, glucose levels were similar, except for a lower increment in glucose levels after 4 min in the EV‐miR‐30c group compared to the EV‐Scr group (Figure S3D, *p* = 0.58 and Figure S3E, *p* = 0.02). No changes in the K_ITT_ were observed either (Figure S3F, *p* = 0.11). These data suggest insulin sensitivity is not modulated by treatment with EV‐miR‐30c.

There was no difference in liver mass between mice treated with EV‐Scr and EV‐miR‐30c mice (Figure 6d, *p* = 0.40). ALT and AST activities in the serum were lower in the mice that received EV‐miR‐30c (Figure 6e,f, *p* = 0.03 and *p* = 0.02, respectively). MTS of liver sections revealed a lower frequency of grade 2 liver fibrosis in mice that received EV‐miR‐30c compared to EV‐Scr (Figure 6g,h, *p* = 0.01). Supporting those histological findings, we evaluated the mRNA expression of fibrogenesis‐related genes (Figure 6i). Among the genes related to fibrogenesis and epithelial‐mesenchymal transition, *Acta2* expression was reduced and e‐cadherin (*Cdh1*) upregulated in the liver of EV‐miR‐30c‐treated mice (Figure 6i, all *p* < 0.05). No differences in *Snai1*, *Col1a1* and *Col3a1*, *Mmp9*, *Mmp12*, *Timp*1 and 3, and *Tgfr1* were found between the groups (Figure 6i, all *p* > 0.05); however, *Timp2* was upregulated in EV‐miR30c livers (Figure 6i, *p* = 0.02). In agreement with *Acta2* gene expression, IF analysis revealed a significantly lower content of α‐Sma in the liver of aged mice treated with EV‐miR‐30c (Figure 6j,k, *p* = 0.04). Vimentin was reduced, but this difference was not statistically significant due to high variability among the mice in the same group (Figure 6j,l, *p* = 0.21). We also quantified the transcript levels of angiogenesis‐related genes and found that *Dll4*, *Angpt2* and *Vegfb* were upregulated in the liver of EV‐miR‐30c‐treated mice; however, *Vegfa* expression was not affected (Figure 6i, *p* = 0.03 for both). The macrophage marker *F4/80* had reduced expression in the liver of the EV‐miR‐30c group compared to the control group (Figure 6i, *p* = 0.03). In addition, mRNA expression of senescence markers and SASP factors was evaluated. *Il1a* and *Il1b* were not differently expressed between the groups, and even though *Tp53* had also similar expression, *Serpine1* and *Cdkn1a* were upregulated in mice that received miR‐30c‐5p (Figure 6i, *p* = 0.03 and *p* = 0.02, respectively).

We measured the expression of miR‐30c in the liver following delivery but found that the levels were similar between the groups (Figure S3G, *p* = 0.78). No differences in miR‐30c‐5p circulating levels were found in both EV and non‐EV fractions (Figure S3H, *p* = 0.55 and Figure S3I, *p* = 0.66). Despite similar levels of miR‐30c between EV‐Scr and EV‐miR‐30c groups, forkhead box O3 (Foxo3), a direct target of miR‐30c‐5p, is significantly reduced in the liver of mice that received miR‐30c (Figure 6m,n, *p* = 0.04). Other proteins that could be involved in the posttranslational modification of Foxo such as members of the epidermal growth factor receptor (Egfr) and phosphatidylinositol 3‐kinase/Akt, and p38 involved in the transcriptional regulation of *Foxo3* were not different in the liver of EV‐miR‐30c compared to the EV‐Scr group (Figure S4A–D, all *p* > 0.05).

Our data shows that only by modifying the content of miR‐30c in EVs from aged mice were we able to decrease the activation of HSCs in the liver similar to the effect of EVs from young mice. Induction of HSCs senescence is a possible mechanism as senescence markers were increased in the liver EV‐miR‐30c‐treated mice. Interestingly, angiogenic factors were induced by the treatment; however, *Timp2*, an inhibitor of angiogenic response was upregulated (Seo et al., 2003). The effect of miR‐30c in the regression of liver fibrosis was not involved with improved insulin sensitivity as no differences were found after the ITT test. Posttranscriptional activation of Foxo3 by miR‐30c‐5p is a plausible mechanism as Foxo3 is a direct target and regulates cellular aging.

## 3 DISCUSSION

The beneficial effect of factors in the blood of young mice on the aging liver has been shown in heterochronic parabiosis experiments. Such experiments increased aged hepatocyte proliferation and restored the CEBPA complex to levels seen in young animals (Conboy et al., 2005).

In this study, we report for the first time that repeated administration of serum EVs from young mice to aged mice improved aged‐related fibrosis, increased locomotion, and whole‐body insulin sensitivity by increasing p‐Akt levels in the skeletal muscle. All these improvements have the potential to slow aging. EVs from exercised mice promoted an extra benefit by also acting in the adipose tissue and liver improving insulin sensitivity as suggested by measurements of mRNA expression of genes related to glucose and fatty acid metabolism and increasing p‐Akt levels in the liver.

The potential of EVs to increase lifespan and promote longer health span has been reported by others (Chen et al., 2024; Grigorian Shamagian et al., 2023; Yoshida et al., 2019). The beneficial effects of young EVs on aged mice have been demonstrated not only on tissues, but also on glucose tolerance, physical activity, cognition, and adiposity (Chen et al., 2024; Grigorian Shamagian et al., 2023; Yoshida et al., 2019). Similarly to this study, Chen et al. (2024) reported an increase in energy expenditure and locomotor activity in aged mice that received EV from young compared to age‐matched control mice treated with PBS. They did not measure food intake; however, the effect may be due to increased food consumption, as we found herein. EVs can cross the blood–brain barrier (Morales‐Prieto et al., 2022) and adipocyte‐derived EVs from obese mice are internalized by proopiomelanocortin neurons, wherein they activate mammalian target of rapamycin signaling and regulate body energy intake (Gao et al., 2020). Therefore, the higher food intake promoted by EV‐C compared to the other groups could be related to an effect in the hypothalamus.

With regards to exercise, acute bouts of exercise have been shown to modulate small EV's protein content involved in the activation of the immune system and NAMPT, which can influence the abundance of NAD^+^, and these changes were found to be dependent on the age and metabolic fitness of the individuals (Chong et al., 2022). Treatment of sedentary mice with EVs derived from the plasma of trained mice improves glucose tolerance, insulin sensitivity, and decreases plasma levels of triglycerides (Castaño et al., 2020). In our study, we have obtained similar findings in aged individuals treated with EV‐EX.

Concerning the liver, we found that EVs from young mice can mitigate aging‐related fibrosis by decreasing HSC activation. Although EVs from sedentary and exercise young mice had the same effect on reducing liver fibrosis, we propose that EV from young per se induces HSC cell senescence as we observed an increase of senescence markers in the liver of EV‐C‐treated mice. On the other hand, EVs from young exercised mice seem to shift intrahepatic balance from inflammation to restoration as we observed an increase in *Mmp12* and a decrease in inflammatory cytokines. EVs from healthy human subjects can reverse liver fibrosis in carbon tetrachloride‐induced hepatic fibrosis independent of gender (Chen et al., 2018). Using PHK67‐labeled EVs, the authors showed that EVs target preferentially HSCs (Chen et al., 2018). Regarding aging, recently, it has been shown that intravenous injection of EVs from young mice into aged mice mitigates senescent phenotypes and ameliorates age‐associated functional declines in multiple tissues (Chen et al., 2024). So far, there is no evidence that EVs from exercised mice reduce liver fibrosis; however, high‐intensity aerobic exercise improves fibrotic and inflammatory conditions of the liver in sedentary obese men with NAFLD (Oh et al., 2017).

Extracellular vesicle cargos contain proteins, nucleic acids, lipids, and metabolites (Chitti et al., 2024). Raman spectroscopy analysis of young and aged EVs revealed no substantial differences in the protein content of EVs according to age; however, aged EVs displayed decreased nucleic acids with a concomitant increase in lipid content (Sahu et al., 2021). Sequencing of small regulatory RNAs in two mouse plasma fractions at five‐time points across the lifespan from two to 18 months revealed that for miRNAs, the EV‐RNA fraction was strongly associated with aging (Kern et al., 2023). Circulating EV‐miRNAs have also been shown to be modulated by high‐intensity interval training and chronic aerobic exercise in lean and obese mice, respectively (Castaño et al., 2020; de Mendonça et al., 2020).

Using expression levels and pathway analysis, we identified circulating EV‐miR‐30c‐5p as a miRNA associated with aging. Profiling of whole blood miRNAs from 3, 8, 12, and 24‐month‐old mice revealed miR‐30c‐5p among the six microRNAs related to liver aging (Kim et al., 2017). Interestingly, in the circulation of eight‐month‐old mice, miR‐30c‐5p was increased compared to three‐month‐old mice; however, it does decrease over time in the circulation and the liver (Kim et al., 2017). While we only compared miR‐30c‐5p expression between two and 18‐month‐old mice, the difference in EV and liver miR‐30c‐5p may be even more pronounced in mice older than 18 months. Herein, we suggest that miR‐30c‐5p is a potential early marker of aging‐related liver fibrosis.

In the liver of EV‐A‐treated mice, miR‐30c‐5p was reduced compared to PBS or EV‐C and EV‐EX. Vimentin, an established target (Suh et al., 2014), was correspondingly increased in those livers. We also suggest that a reduction of miR‐30c‐5p expression in the liver of aged mice is a contributing factor to worsening liver fibrosis. Downregulation of miR‐30c‐5p has also been observed in hepatitis B‐related fibrotic patients (Gu et al., 2021) and shown to promote hepatocarcinoma progression (He et al., 2021). Of note, VEGF has been shown to reduce expression of miR‐30c‐5p (Gu et al., 2021) and, *Vegfa* levels were reduced in the liver of aged mice treated with young EVs.

A protective effect of EV‐miR‐30c in liver fibrosis was observed in our study, as evidenced by reduced activation of HSCs indicated by α‐Sma content. Our study agrees with Chen et al., 2018, who showed that HSCs are one of the main target cells of EVs (Chen et al., 2018). Even though angiogenesis was activated, Timp‐2, which has been previously shown to inhibit Vegf‐induced angiogenic response (Seo et al., 2003) was upregulated and potentially protects from advanced liver fibrosis over time.

Despite the fact that miR‐30c‐5p levels did not change in the liver, Foxo3, a previously validated target (Jeon et al., 2015; Li et al., 2018), was significantly reduced, which appears to confirm that miR‐30c‐5p was delivered. Foxo3 reduction in the cytoplasm was not mediated by Egfr/Pi3k/Akt or p38 as their levels were similar between the EV‐miR‐30c and EV‐Scr groups. However, the FOXO family is subjected to other covalent modifications such as acetylation, methylation, and ubiquitination and we cannot discard the possibility that other inhibitors are involved in *Foxo3* reduction.

*FOXO3* has been shown in many studies to be increased in liver fibrogenesis, in activated HSCs both in vitro and in vivo, and also in livers of patients with hepatocarcinoma (Kim et al., 2020; Li et al., 2015; Zhu et al., 2018). In HSCs, Foxo3 activation is related to amplification of the Tgf‐β/Smad signaling pathway, facilitating fibrotic gene expression (Kim et al., 2020).

Similarly to the treatment with EV‐C, senescent cell markers were significantly upregulated in the liver of EV‐miR‐30c mice and may be related to the fact that inhibition of *FOXO3* was associated with enhanced cellular senescence in human fibroblasts (Xie et al., 2014). HSCs senescence is one of the mechanisms that may limit and reduce fibrosis (Kisseleva & Brenner, 2020).

One possible limitation of our study is the fact that we could not detect in single‐cell resolution the expression of miR‐30c‐5p and track the delivered EV‐miRNA by using labeled miRNAs due to the limitation with dilution of the labeled mimic in vivo and the possibility that when the miRNA is processed into Argonaute (Ago), it loses the label.

In conclusion, our data suggest that EVs from young individuals when delivered to aged subjects protect them from developing advanced liver fibrosis as observed with EV‐A by reducing the activation of HSCs. The mechanism of the fibrosis regression seems to differ between EV‐C and EV‐EX. While EV‐C potentially induces HSC senescence, EV‐EX reduces inflammation and accelerates elastin degradation by inducing *Mmp12*. The hepatic levels of miR‐30c‐5p were correlated with HSC activation as shown by the protective effect of EV‐miR‐30c in liver fibrosis. Similar to EV‐C, senescent markers were upregulated and angiogenesis response was inhibited in the liver of EV‐miR‐30c‐treated mice. Because miR‐30c treatment was performed with EV from aged mice the difference between EV‐A in model 2 and model 3 is the amount of miR‐30c within the EV. Therefore, we may suggest that miR‐30c is the mediator of the deactivation of HSC induced by EVs from the young and possibly induces HSC senescence. Additionally, exercise may have other factors in the EV contributing to liver fibrosis resolution that need to be addressed in future studies.

Aging is the highest risk factor for many diseases. Although humans are living longer, they are not necessarily living healthy. Strategies that promote longevity and improve health are desired. EV‐encapsulated miR‐30c‐5p could be a potential co‐adjuvant therapy in reducing aging‐induced liver fibrosis.

## 4 MATERIALS AND METHODS

### 4.1 Animals

All animals (two‐month‐old and 18‐month‐old C57BL/6 male mice) were obtained from the National Institute of Aging (NIA) colonies. Mice were housed in ventilated cages (2–4 animals/cage) at the Animal Research Facility, Center for Life Sciences, Beth Israel Deaconess Medical Center (BIDMC). The mice were maintained at 12:12‐h light–dark cycles, 22°C ± 2°C room temperature (RT), received LabDiet 5008 standard diet of 3.56 kcal/g (Land O'Lakes Inc., Arden Hills, MN) and water ad libitum all through the experimental period. Cages and bedding were changed once every 2 weeks. Mice were monitored regularly for their health status by animal technicians with the support of veterinarians, and remained free of any adventitious infections for the entire duration of this study. The BIDMC Institutional Animal Care and Use Committee approved this study (15/2022).

## 5 EXPERIMENTAL MODELS

### 5.1 Model 1: EVs isolation

Two‐month‐old (“young”, *n* = 30) and 18‐month‐old mice (“aged”, *n* = 26) mice were ear tagged and randomly assigned to the following groups: young sedentary (C), young exercised (EX), and aged (A). After 1 week of their arrival at the animal facility, their body weight was measured. An insulin tolerance test and EchoMRI were performed that week. In the second week, mice in the EX group were subjected to acute treadmill exercise while mice in C or A groups were left in the cage for 90 min between 9 AM and 10:30 AM. Immediately after, mice were euthanized (between 10:30 AM and 11:30 AM). After euthanasia, cardiac blood was collected for serum separation followed by EV isolation. The EVs collected from each group were labeled as EV‐C (from sedentary mice), EV‐EX (from exercised mice), and EV‐A (from aged mice).

### 5.2 Model 2: Homologous and heterologous transfer of EVs

Eighteen‐month‐old mice (“aged,” *n* = 31) were randomly intravenously injected with phosphate buffer saline (PBS) obtained from the void of the column (*n* = 9), EV‐A (*n* = 7), EV‐C (*n* = 9), or EV‐EX (*n* = 6) once a week for 4 weeks. Body weight and composition, ITT, and indirect calorimetry were performed after the last injection. Five weeks after the beginning of the first EV transfer, mice were euthanized. Gastrocnemius skeletal muscle, visceral and subcutaneous fat depots, and liver were dissected and weighed. A portion of fat depots, gastrocnemius, and liver were flash frozen in liquid nitrogen and stored at −80°C until RNA and/or protein extraction, and the remainder of the tissues were fixed in formalin for histological analysis. The blood was used for serum separation and kept at −80°C until use for hepatic enzyme measurements. Three experimental cohorts were performed with 3–4 mice per group.

### 5.3 Model 3: EVs loaded with miR‐30c‐5p mimic

Fourteen aged mice (*n* = 7/group) were ear tagged and randomly assigned to the following groups: mice that received EVs transfected with scrambled miRNA sequence (EV‐Scr), and mice that received EVs transfected with miR‐30c‐5p mimic (EV‐miR‐30c). Mice were intravenously injected with the corresponding EVs once a week for 4 weeks. Body weight and composition, ITT, and indirect calorimetry were performed after the last injection. After 5 weeks since the beginning of the EV transfer, mice were euthanized. Blood was collected, visceral and subcutaneous fat depots, and liver were dissected and weighed. A portion of the liver was flash‐frozen in liquid nitrogen and stored at −80°C until RNA/protein extraction, and the rest was fixed in formalin. The blood was used for serum separation. Serum was stored at −80°C until EV isolation and determination of hepatic enzymes activities. One experimental cohort was performed with 7 mice per group.

### 5.4 EVs isolation and characterization

Extracellular vesicle suspensions were obtained using size exclusion chromatography via the qEV isolation platform (Izon Science, Medford, MA). EVs used for the treatment of aged mice were obtained as follows: approximately 1 mL pre‐cleared serum from 3 to 5 animals from each group was loaded in 35 nm qEV1 columns (Izon Science) according to the manufacturer's instructions. Briefly, samples were pre‐cleared by two‐step centrifugation: centrifuge at 2000 × *g* for 10 min at RT, and then centrifuge at 10,000 × *g* for 15 min at RT. Fractions six through nine were collected. The purified EVs of the same group were pooled. The EV suspensions were then concentrated by an Amicon ultra‐2 mL centrifugal filter device (Millipore, Burlington, MA) and stored at −80°C. A portion of the EV suspension was quantified by nanoparticles tracking and characterized using western blotting of EV markers (CD9, CD81, and Alix) and EV contaminants (apoA1 and B) (see later section).

Another set of EV suspension was obtained for miRNA profiling (see later section). EV suspensions were obtained from 150 μL of serum from each mouse (*n* = 6–10/group). After two‐step centrifugation, serum was loaded into a qEVsingle/35 nm Gen 2 column to obtain EV suspensions as described above. EVs were concentrated using the qEV concentration Kit (Izon Science), and RNA was immediately extracted.

### 5.5 Nanoparticle tracking analysis

The size distribution and particle concentration were measured using the Nanosight NS300 instrument (Malvern Instruments Ltd., Amesbury, UK) with a 488 nm laser, a high‐sensitivity sCMOS camera, and a syringe pump. All samples were diluted 1:200 in fresh‐filtered PBS using the following settings: camera level 11 and detection threshold 5. Experiment videos were analyzed using the Nanoparticle Tracking Analysis 3.4 Build 3.4.003 after capture in script control mode (three videos of 30s per measurement) using syringe pump speed 100. A total of 749 frames were examined per sample.

### 5.6 EV administration

Extracellular vesicles (10^9^ particles in 100 μL of PBS) were used to treat aged mice intravenously, once a week for 4 weeks. Injections were administered between 10:00 AM and 12:00 PM by the same operator.

### 5.7 Transfection of EVs loaded with miR‐30c‐5p mimic or scramble

Extracellular vesicles were isolated from the serum of aged mice and mixed in a pool to ensure consistency. A solution containing 2 × 10^9^ EVs was transfected with 150 pmol of scramble (*negative control*) or 150 pmol of miRCURY LNA miR‐30c‐5p (Qiagen, Germantown, MD) by using the Exo‐Fect™ Exosome Transfection Reagent (*SBI* System Biosciences, Palo Alto, CA) following the manufacturer's instructions. Each EV pellet was resuspended in 300 μL of PBS, which was enough for two i.v. injections; thus, four preparations were freshly prepared every week on the day of transfection and immediately injected into the mouse tail vein.

### 5.8 ITT

After a five‐hour food restriction, mice received an intraperitoneal injection of a dose of 0.75 UI/kg body weight insulin (Novolin® R, Novo Nordisk, Bagsvaerd, Denmark). Glucose levels were measured using a glucometer (Accu‐Chek Guide, Roche, Indianapolis, IN) by capillary blood caudal collection before (i.e., 0 min) and 4, 8, 12, 16, and 20 min after insulin administration. As an estimation of insulin action, we calculated the plasma glucose disappearance rate (K_ITT_), represented by the angular coefficient (slope) of the linear phase of glucose decay; in our study between four and 16 min after insulin administration. We also calculated the area under the curve (AUC) of the glucose curve obtained.

### 5.9 Body composition and indirect calorimetry

The body composition of the animals was evaluated by EchoMRI 3‐in‐1 body composition analyzer (EchoMRI, Houston, TX). For measurements of metabolic rate and food intake, approximately 19‐month‐old mice treated with EVs or PBS and fed standard diet were placed within the Comprehensive Lab Animal Monitoring System (CLAMS) (Columbus Instruments, Columbus, OH). Rates of oxygen consumption (VO_2_) and CO_2_ production (VCO_2_) were determined from gasometric sensors. These data were used to calculate the respiratory exchange rate (RER: RER = VCO_2_/VO_2_) and energy expenditure (EE = (3.815 + 1.232 × RER) × VO_2_). Respiratory exchange ratio (RER) results for two mice were excluded because of equipment malfunction. Energy balance was calculated as the difference between total food intake (Kcal) and EE (Kcal). All calculations and analysis were performed using CalR, an online tool, with the ‘remove outliers’ feature activated (Mina et al., 2018).

### 5.10 Serum ALT and AST enzyme activities

Cardiac blood was allowed to clot for 30 min at room temperature and then centrifuged at 1500 × *g* for 10 min for serum separation. Serums with high grades of hemolysis were not used for the analysis. ALT and AST enzyme activities were measured using ALT/GPT and AST/GOT liquid reagents following the manufacturer's instructions with minor modifications (catalog number: A524 and A559, respectively, Teco Diagnostics, Anaheim, CA). For each test, 10 μL of serum was used and mixed with 100 μL of working reagent freshly prepared. The samples were run in duplicate. Absorbance at 340 nm was read in a SpectraMax® iD3 spectrophotometer (Molecular Devices, San Jose, CA).

### 5.11 Proteome profiler

Liver from young mice and aged mice treated with EV‐A or EV‐C or EV‐EX were pulverized in liquid nitrogen then 20 mg of each liver sample was homogenized in PBS with cOmplete™ mini protease inhibitor cocktail (Roche Diagnostics Gmbh, Mannheim, Germany) and 1% Triton X‐100. Samples were frozen at −80°C, thawed, and centrifuged at 10,000 × *g* for 5 min at 4°C to remove cellular debris. The amount of total protein amount was quantified using the bicinchoninic acid (BCA) kit (Thermo Scientific, Waltham, MA) and immediately assayed using a Proteome Profiler Mouse XL Cytokine Array kit (Catalog number: ARY028, R&D systems, Minneapolis, MN) that simultaneously profiles 111 cytokines. Chemiluminescence was detected by using the Clarity Western ECL substrate (Bio‐Rad, Waltham, MA) in a GE Amersham Imager 600 Imager (GE Healthcare Bio‐Sciences, Piscataway, NJ). Pixel densities on developed membranes were collected using Image Studio Lite version 5.2 (LI‐COR Biosciences, Lincoln, NE). Signals on different arrays were compared to determine the relative change in analyte levels between samples. Aged mouse treated with EV‐A was used as the reference.

### 5.12 Western blotting

Liver tissue was pulverized in liquid nitrogen, and 50 mg was homogenized in Radio‐Immunoprecipitation Assay (RIPA) buffer (Sigma‐Aldrich, St. Louis, MO) with cOmplete™ mini protease inhibitor cocktail using a tissue homogenizer, and then sonicated for 1 min, 6 pulses for 6 s on/off (Sonicator Ultrasonic Processor, Heat Systems). Nuclear extracts were obtained by cell fractionation using the NE‐PER reagent (Thermo Scientific). Total protein was determined using the BCA protein array kit then 20 μg of total protein was mixed with 4× Laemmli buffer (Bio‐rad), boiled for 5 min at 95°C before loading in a Novex gradient 4%–12% Bis‐Tris polyacrylamide gel (Thermo Scientific) for electrophoresis.

For EVs, 10 μL of concentrated EV‐A, EV‐C and EV‐EX were first mixed with RIPA buffer at 1:1 vol, then further combined with 4× Laemmli buffer before loading in the same 4%–12% polyacrylamide gel.

After the electrophoresis run was complete, proteins were transferred onto a nitrocellulose membrane using the iBlot2 system (Thermo Scientific). Anti‐ApoA1 (catalog# PA5‐29557, Invitrogen, Waltham, MA), anti‐ApoB (20578‐1‐AP, Proteintech, Rosemont, IL), anti‐Alix (#2171, Cell Signaling technology (CST), Danvers, MA), anti‐CD81 (#10037, CST), anti‐CD9 (C9993, Sigma‐Aldrich), anti‐Akt (#2920, CST), anti‐phospho‐Akt (Ser473) (#4060, CST), anti‐GAPDH (#2118, CST), anti‐IkBα (#4812, CST), anti‐NF‐kBp65 (#8242, CST), anti‐phospho‐NF‐kBp65 (Ser536) (#3033, CST), anti‐FoXO3a (#12829, CST), anti‐phospho‐p38 MAPK (Thr180/Tyr182) (#4511, CST), anti‐p38 MAPK (#8690, CST), anti‐EGFR (#4267, CST), anti‐phospho‐EGFR (Tyr1068) (#2234, CST) were diluted 1/1000 or 1/2000 in Tris Buffered Saline with Tween 20 (TBS‐T; 20 mM Tris, 150 mM NaCl, pH 7.5, 0.1% Tween‐20) supplemented with 5% bovine serum albumin (BSA) or 5% non‐fat milk according to the manufacturer's instructions and visualized by blotting with horseradish peroxidase (HRP)‐conjugated secondary anti‐rabbit antibody (#7074, CST) or anti‐mouse (#7076, CST) 1:2500 dilution in 3% non‐fat milk (#7076, CST). Chemiluminescence was detected by using the Clarity Western ECL substrate in a GE Amersham Imager 600 Imager.

### 5.13 Profiling of miRNAs in EVs

The ID3EAL™ Biofluid miRNA Knowledge Panel v.3.9 (Mirxes Pte Ltd., Singapore) simultaneously profiles 176 human miRNAs per sample using real‐time quantitative PCR (qPCR) technology. There are 109 mouse homologs among the 176 human miRNAs. This assay is in a 384‐well plate format and can accommodate two samples per plate. Total RNA from 24 concentrated EVs samples was extracted using qEV RNA extraction kit (Izon Science). For each sample, 100 ng was used to perform reverse transcription using the ID3EAL™ miRNA RT kit (Mirxes) adhering to the manufacturer's protocol. ID3EAL™ miRNA qPCR master mix was used for the qPCR reactions and performed using the QuantStudio 3 Real‐time PCR system (Thermo Scientific) with the amplification protocol: 1 cycle of 95°C for 10 min, 40°C for 5 min, followed by 40 cycles of 95°C for 10 s and 60°C for 30 s. These assays were carried out jointly with the Detection Unit, Precision RNA Medicine Core at BIDMC.

Raw cycle threshold (*C*_t_) data were combined into a data matrix for processing into analysis‐ready format using R. The *C*_t_ for undetectable miRNAs was coded as 40. Any miRNAs with raw *C*_t_ ≤ 9 or ≥33 were initially excluded. *C*_t_ values of the two RNA spike‐ins and the two inter‐plate calibrators were inspected and used to correct for intra‐run variations. Next, we imputed the mean ± 3 standard deviation (SD) value for initially excluded miRNAs whose raw *C*_t_ were ≤9 or ≥ 33. This allows global normalization to be performed. ComBat (SVA package, R) was used to adjust for batch effects as miRNA profiling for 24 samples was performed across 5 days. Each miRNA was now expressed as Δ*C*_t_.

### 5.14 Quantification of mRNA in tissues

Total RNA from liver and inguinal fat was extracted using Trizol (Thermo Scientific) according to the manufacturer instructions. Reverse transcription was performed using 500 or 2000 ng of total RNA from fat tissue and liver, respectively, using Superscript IV (Thermo Scientific) and Random Primers (Thermo Scientific). The cDNA obtained was diluted 10× in nuclease‐free water and stored at −20°C. Primers for each gene of interest were designed using NCBI BLAST tools and purchased from Integrated DNA Technologies (IDT) (Table S1). Primers for *pri‐mir‐30c‐1* (Mm03306692_pri) and *pri‐mir‐30c‐2* (Mm03306701_pri) were purchased from Thermo Scientific. Relative quantification of mRNA was performed by qPCR with detection by the LightCycler® 480 SYBR Green I Master except for the pri‐mirs that we used LightCycler® 480 RNA Master hydrolysis probes (Roche Diagnostics), using the Roche LightCycler II 480 machine. *Hprt1* and *Gapdh* were used as reference genes as they were the most stable of the six housekeeping genes tested (*Ppia*, *Rpl0*, *Rpl19*, *Srf4*, *Hprt1*, and *Gapdh*) as indicated by Normfinder (MOMA, Aarhus, Denmark).

### 5.15 Quantification of miRNA in tissues and EVs

The relative quantification of the miR‐30 family (miR‐30a‐5p, miR‐30b‐5p, miR‐30c‐5p, miR‐30d‐5p, and miR‐30e‐5p) and housekeeping small RNAs *Snord110/Snord68* and miR‐103a‐3p was performed by qPCR using the miRCURY LNA™ miRNA system (Qiagen). cDNA synthesis was performed from 10 ng of total RNA obtained from EVs or liver using the miRCURY LNA RT kit (Qiagen). The reagents were mixed and incubated in a thermocycler for 60 min at 42°C and 5 min at 95°C. The cDNA was further diluted 60× in nuclease‐free water and stored at −20°C. MiRNA primers for miR‐103a‐3p (GeneGlobe ID YP00204063), *Snord68* (GeneGlobe ID YP00203911), *Snord110* (GeneGlobe ID YP00203912), miR‐30c‐5p (GeneGlobe ID YP00204783), miR‐30a‐5p (GeneGlobe ID YP00205695), miR‐30b‐5p (GeneGlobe ID: YP00204765), miR‐30d‐5p (GeneGlobe ID YP00206047) and miR‐30e‐5p (GeneGlobe ID YP00204714) were purchased from Qiagen. The expression levels of the miRNAs were revealed by qPCR with detection by miRCURY LNA SYBR Green kit (Qiagen), using the Roche LightCycler II 480 machine and following the universal amplification protocol: 95°C for 10 min followed by 40 cycles of 95°C for 15 s and 60°C for 1 min, followed by a dissociation curve. For the quantification of gene expression, the relative quantification method was used using the constitutive genes *Snord110* and *Snord68* as references for tissues and miR‐103a‐3p for serum EVs using the $2^{-∆∆C_{t}}$ method. These genes were chosen as reference genes as their expression was not affected by treatment or age.

### 5.16 Histological analysis

Mouse liver was fixed overnight in 10% formalin and embedded in paraffin for sectioning and H&E staining, MTS, immunofluorescence (IF), and immunohistochemistry (IHC). Histology, IF, and IHC were performed by the Histology Core Facility at BIDMC, and visualized using the Axioimager microscope (Carl Zeiss, White Plains, NY). Image acquisition was performed using the AxioCamHR and AxioCamMR for brightfield and fluorescence imaging, respectively. For MTS, the fibrosis score ranged from zero to four and was blindly scored with the help of Dr. Roderick Bronson, Rodent Pathology Core at Harvard Medical School. The following criteria were used to define the grade of fibrosis: Grade zero: no fibrosis or scarring; Grade one: minimal fibrosis mild scarring; Grade two: moderate fibrosis or scarring that extends outside the liver; grade three: advanced fibrosis or severe scarring; and grade 4: severe fibrosis or cirrhosis. IF was performed for the following antibodies: α‐smooth actin (α‐SMA) (A5228, Sigma, 1:1000), Vimentin (ab92547, Abcam, 1:400) and for nuclei staining Hoescht 33,342 10 mg/mL solution (Thermo Scientific) was used. The percentage area of MTS, α‐SMA, and vimentin staining were calculated using ImageJ.

### 5.17 Statistical analysis

Data are presented as mean ± SD. For two groups, the means were compared using Student's *t* test. For three or more groups, one‐way ANOVA was used to test for statistical significance. For ITT blood glucose levels and body weight in different time points, two‐way ANOVA was used. A *p*‐value of <0.05 was considered significant. Tukey's *post‐hoc* test was used for pairwise comparisons. The *p* value of the *t* test or one‐way ANOVA is generally reported in the Results section unless otherwise stated. GraphPad prism 8.0 version 2.1 was used to plot graphs and for statistical analysis. Indirect calorimetry analysis was performed using the CalR online tool as explained in detail by Mina et al., 2018. Food and water intake, energy expenditure, oxygen, and carbon dioxide consumption were analyzed by ANCOVA/generalized linear models (GLM) using the total mass as a covariate. For measurements not associated with mass (locomotor activity, distance traveled, RER, and energy balance), differences between groups were analyzed by one‐way ANOVA.

For miRNA profiling data, we focused on the 109 mouse homologs miRNAs. Multivariable linear regression was used to compare between the groups, adjusting for plate as a co‐variable since there were two samples per plate. Multiple hypothesis correction was done using the Benjamini–Hochberg method, with a false discovery rate (FDR) of <0.05 as significant. The $2^{-∆∆C_{t}}$ method was used to calculate fold change for each miRNA comparing EV‐C and EV‐EX relative to EV‐A or EV‐EX relative to EV‐C. The log_2_‐transformed fold change was used to construct the heatmap and the volcano plots.

Fold‐changes between EV‐C and EV‐A were used to input into the Ingenuity Pathway Analysis (IPA) software (Qiagen). MiRNAs with a fold change ≤−2.0 and ≥2.0 were selected for comparison analysis and pathway/disease predictions. IPA microRNA target filter tool was used to identify mRNA targets of miR‐30c‐5p and predict the pathways involved.

## AUTHOR CONTRIBUTIONS

Conceptualization, A.C.R. and F.J.S.; Methodology, A.C.R. and F.J.S.; Investigation, A.C.R.; Forma Analysis: A.C.R., Y.J.H.; Writing – Original Draft, A.C.R.; Writing – Review & Editing, A.C.R., Y.J.H. and F.J.S.; Funding Acquisition, F.J.S.; Resources, Y.J.H. and F.J.S.; Supervision, F.J.S.

## CONFLICT OF INTEREST STATEMENT

The authors declare no conflict of interest.

## DATA AVAILIABILITY STATEMENT

Data are available on request from the corresponding authors.

## Supporting information


Figure S1.

Figure S2.

Figure S3.

Figure S4.

Table S1.

Table S2.

Table S3.

Table S4.
