# Supplementary material for: Extracellular vesicle‐encapsulated miR‐30c‐5p reduces aging‐related liver fibrosis
Source: Aging Cell. 2024 Sep 13;23(12):e14310. doi: 10.1111/acel.14310 (PMC11634720; doi:10.1111/acel.14310)
Supplement: Supplementary file 1 — Figure S1. [file ACEL-23-e14310-s002.pdf]

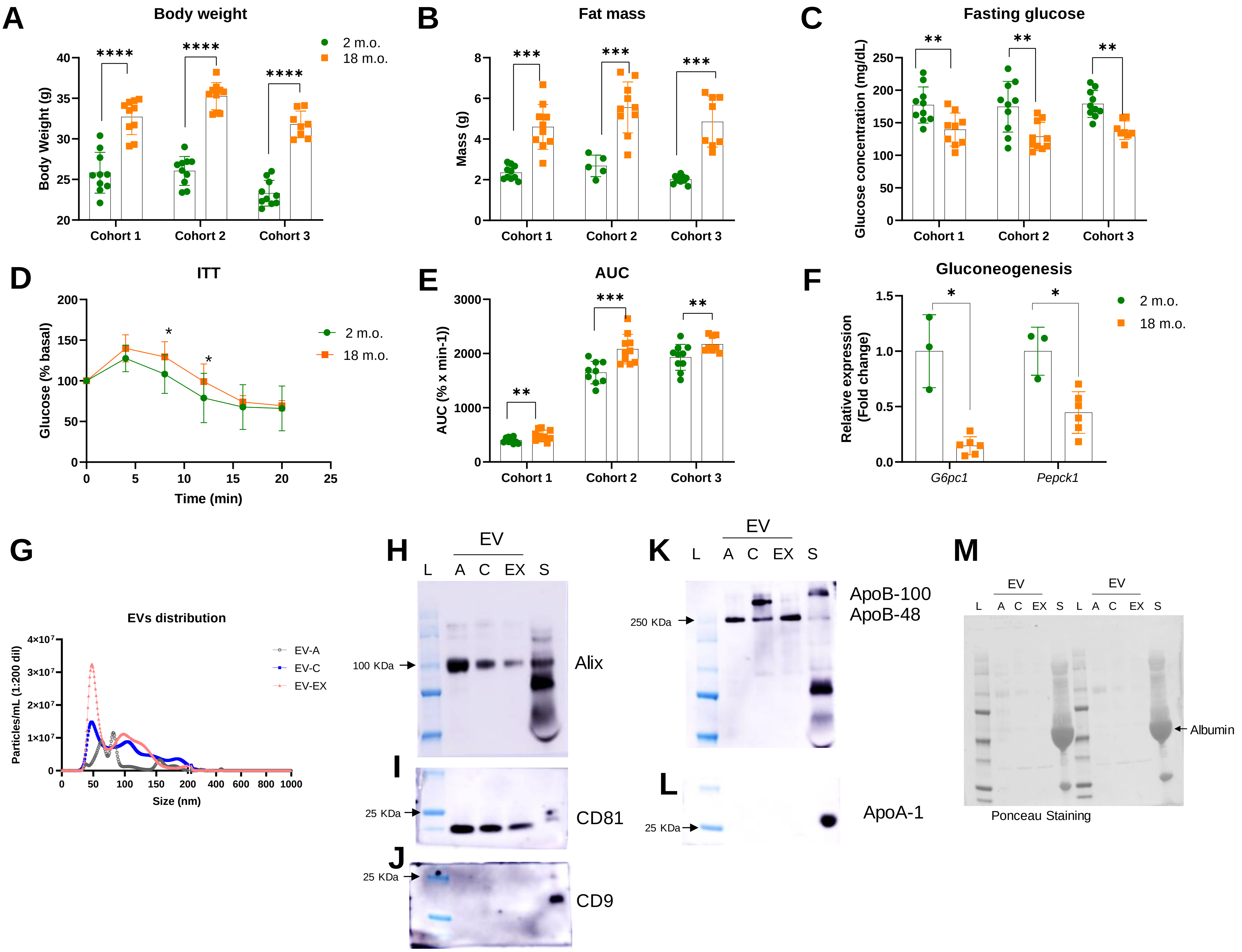
A
Body weight
Body Weight (g)
2 m.o.
18 m.o.
Cohort 1
Cohort 2
Cohort 3
B
Fat mass
Mass (g)
C
Fasting glucose
Glucose concentration (mg/dL)
D
ITT
Glucose (% basal)
Time (min)
E
AUC
AUC (% x min-1))
F
Gluconeogenesis
Relative expression (Fold change)
G6pc1
Pepck1
G
EVs distribution
Particles/mL (1:200 dil)
EV-A
EV-C
EV-EX
Size (nm)
H
EV
L A C EX S
100 KDa
Alix
I
25 KDa
CD81
J
25 KDa
CD9
K
250 KDa
ApoB-100
ApoB-48
L
25 KDa
ApoA-1
M
Albumin
Ponceau Staining

Figure S1: Aged mice are obese and insulin resistant. A-E: Three experimental cohorts comparing 18- month-old (m.o.) and 2 m.o. mice were performed. **A:** Body weight; **B:** fat mass calculated using EchoMRI machine; **C:** Fasting glucose measured with glucose strips after a 5h fast; **D:** Insulin tolerance test (ITT) after a 5h fast, and **E:** Area under the curve (AUC) calculated from ITT. **F:** Gluconeogenesis-related genes were measured in the liver of two m.o. and 18 m.o. mice by qPCR (n=3 and n=6, young and aged, respectively). **p<0.01, ***p<0.001 and ****p<0.0001 as indicated by t test. **G-M:** Extracellular vesicle (EV) characterization. Serum was obtained from 18 m.o. (EV-A), two m.o. sedentary (EV-C) and two m.o. acutely exercised mice (EV-EX). For each group, serum was pooled in 1 mL and then EV was isolated using qEV1 columns. **G**: Representative graph of the distribution of EV by size and concentration measured with a nanoparticle tracking analyzer (NTA). Samples were diluted 1:200 n=1/group); **H-L:** Western blotting of EVs and total serum. Bands obtained after western blotting for Alix (**H**), Cd81 (**I**), Cd9 (**J**), Apob (**K**) and Apoa1(**L**); **M:** Ponceau staining of the membrane used for the blot. Arrow indicates albumin and no albumin was detected in the EVs suspension. L= ladder; A = EV-A; C= EV-C; EX=EV-EX; S = serum.
