# Supplementary material for: Extracellular vesicle‐encapsulated miR‐30c‐5p reduces aging‐related liver fibrosis
Source: Aging Cell. 2024 Sep 13;23(12):e14310. doi: 10.1111/acel.14310 (PMC11634720; doi:10.1111/acel.14310)
Supplement: Supplementary file 2 — Figure S2. [file ACEL-23-e14310-s001.pdf]

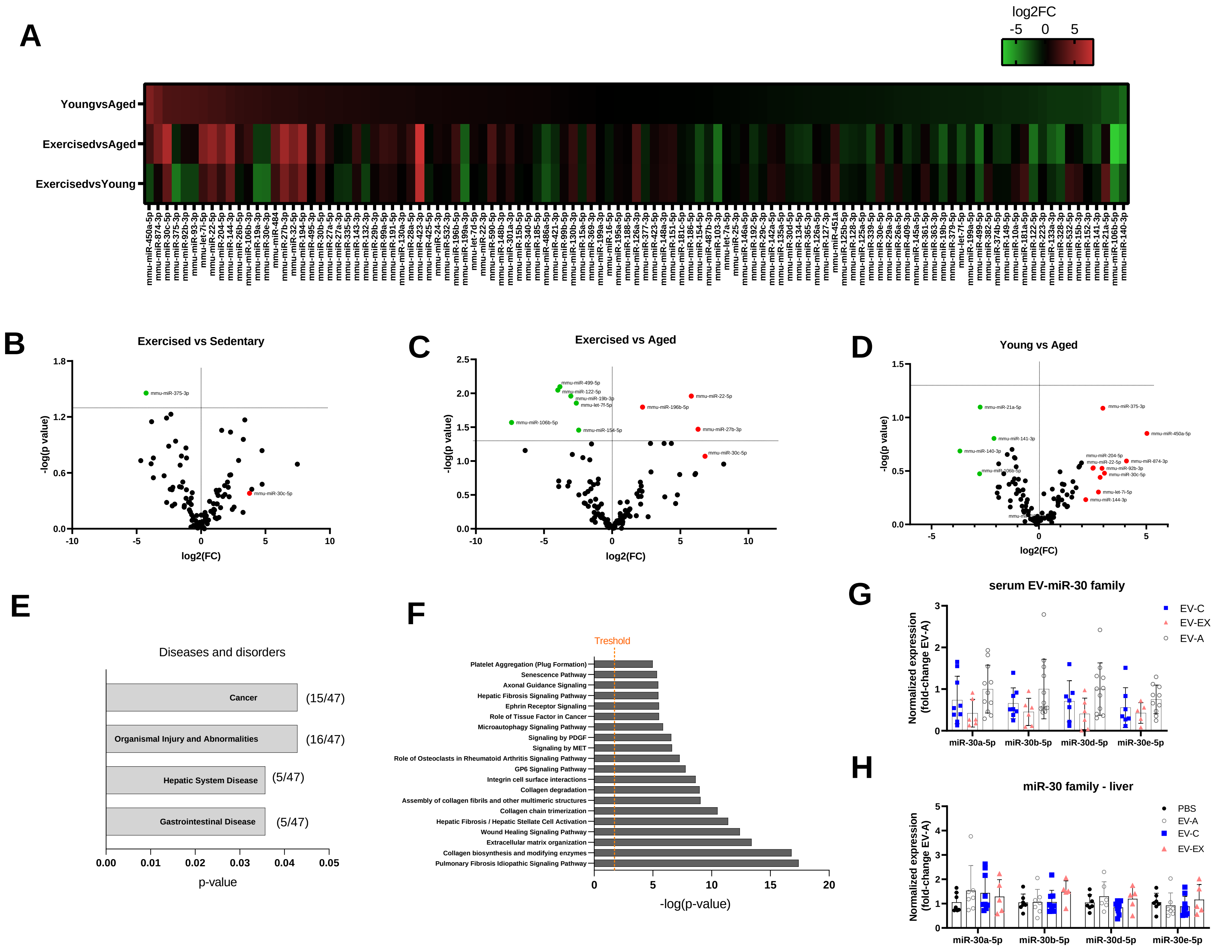
A
log2FC
-5 0 5
YoungvsAged
ExercisedvsAged
ExercisedvsYoung
B
Exercised vs Sedentary
-log(p value)
log2(FC)
C
Exercised vs Aged
-log(p value)
log2(FC)
D
Young vs Aged
-log(p value)
log2(FC)
E
Diseases and disorders
Cancer
(15/47)
Organismal Injury and Abnormalities
(16/47)
Hepatic System Disease
(5/47)
Gastrointestinal Disease
(5/47)
p-value
F
Treshold
Platelet Aggregation (Plug Formation)
Senescence Pathway
Axonal Guidance Signaling
Hepatic Fibrosis Signaling Pathway
Ephrin Receptor Signaling
Role of Tissue Factor in Cancer
Microautophagy Signaling Pathway
Signaling by PDGF
Signaling by MET
Role of Osteoclasts in Rheumatoid Arthritis Signaling Pathway
GP6 Signaling Pathway
Integrin cell surface interactions
Collagen degradation
Assembly of collagen fibrils and other multimeric structures
Collagen chain trimerization
Hepatic Fibrosis / Hepatic Stellate Cell Activation
Wound Healing Signaling Pathway
Extracellular matrix organization
Collagen biosynthesis and modifying enzymes
Pulmonary Fibrosis Idiopathic Signaling Pathway
-log(p-value)
G
serum EV-miR-30 family
Normalized expression (fold-change EV-A)
EV-C
EV-EX
EV-A
miR-30a-5p
miR-30b-5p
miR-30d-5p
miR-30e-5p
H
miR-30 family - liver
Normalized expression (fold-change EV-A)
PBS
EV-A
EV-C
EV-EX
miR-30a-5p
miR-30b-5p
miR-30d-5p
miR-30e-5p

Figure S2: EVs from aged and young mice differ in miRNA content. Serum EVs were isolated from two-month-old (2 m.o) sedentary (EV-C) or acutely exercised (EV-EX) and aged mice (18 m.o.) (EV-A) and profiled for 176 human miRNAs. **A**: Heatmap displayed the Log2-transformed fold change (FC) for each comparison where either aged or young was the reference. **B-D**: Volcano plots for young exercised versus aged, young exercised versus young sedentary and young versus aged. In green down-regulated miRNAs and in red up-regulated miRNAs. Horizontal and vertical broken lines in the volcano plots are the threshold for *p*-value and FC, respectively. **E and F**: Ingenuity Pathway Analysis (IPA). **E**: Disease and disorders associated with the miRNAs with a FC of |2.0| between young and aged EVs; **F**: top 20 statistically significant pathways predicted for miR-30c-5p targets. **G**: miR-30 family member levels in the circulating EVs measured by qPCR. **H**: Expression of other members of miR-30 family in the liver of aged mice treated with PBS or EV-C, EV-EX or EV-A measured by RT-qPCR.
