# Supplementary material for: Extracellular vesicle‐encapsulated miR‐30c‐5p reduces aging‐related liver fibrosis
Source: Aging Cell. 2024 Sep 13;23(12):e14310. doi: 10.1111/acel.14310 (PMC11634720; doi:10.1111/acel.14310)
Supplement: Supplementary file 3 — Figure S3. [file ACEL-23-e14310-s005.pdf]

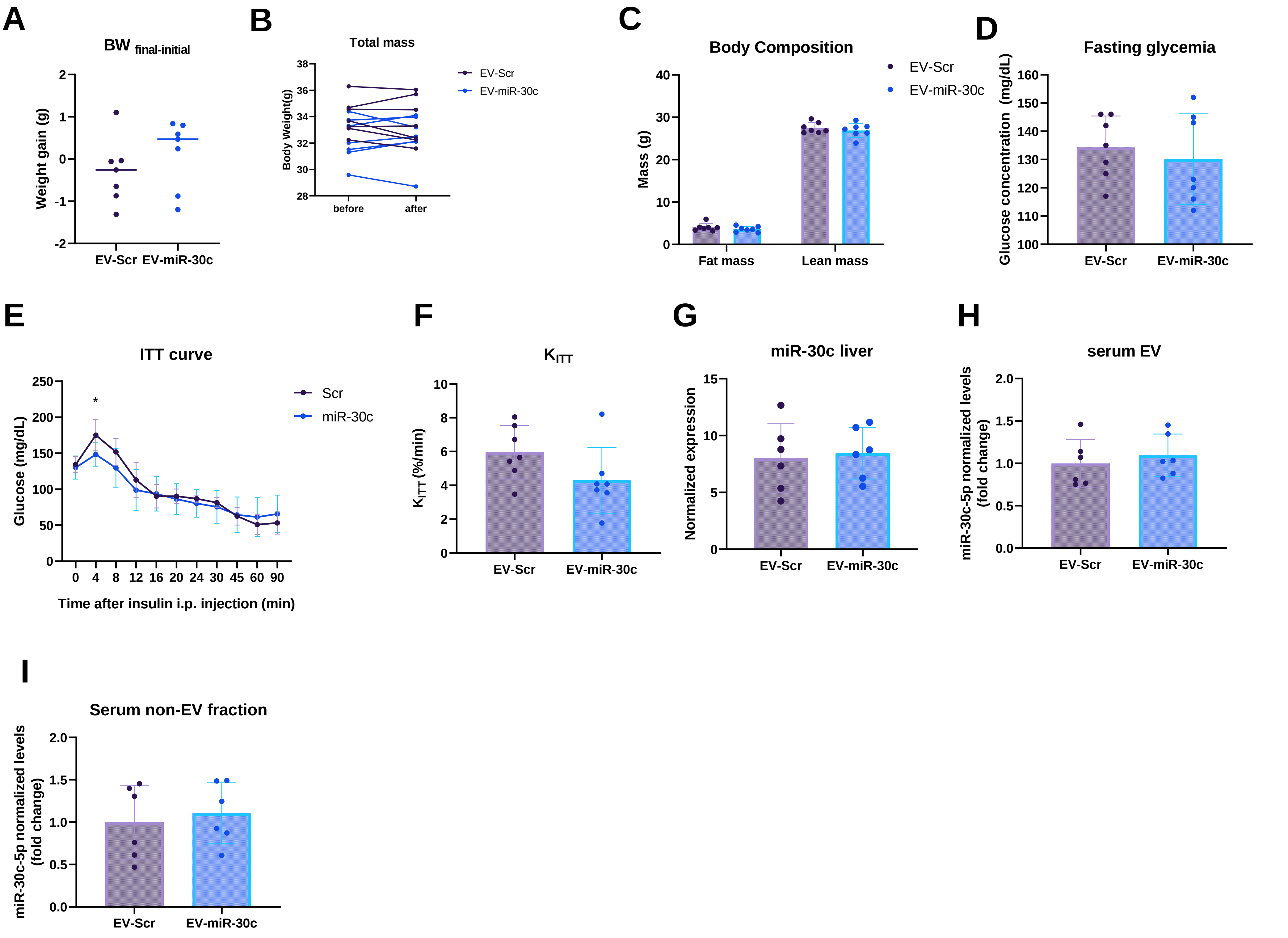
A
BW final-initial
Weight gain (g)
EV-Scr
EV-miR-30c
B
Total mass
Body Weight(g)
before
after
EV-Scr
EV-miR-30c
C
Body Composition
Mass (g)
Fat mass
Lean mass
D
Fasting glycemia
Glucose concentration (mg/dL)
E
ITT curve
Glucose (mg/dL)
Scr
miR-30c
Time after insulin i.p. injection (min)
F
KITT
KITT (%/min)
G
miR-30c liver
Normalized expression
H
serum EV
miR-30c-5p normalized levels (fold change)
I
Serum non-EV fraction
miR-30c-5p normalized levels (fold change)

Figure S3: Therapy with extracellular vesicle-encapsulated miR-30c-5p (EV-miR-30c) does not affect body composition, weight, or insulin sensitivity. Treatment of aged mice with i.v. injections of EV-miR or EV-Scr (negative control) for 4 weeks. **A-C**: body weight gain, body weight before and after treatment and body composition of treated mice. **D:** fasting glycemia; **E**: Insulin tolerance test (ITT) curve; **F:** glucose decay constant (K_{ITT}) calculated from ITT curve; **G-I**: miR-30c-5p levels in the liver, serum EV fraction and non-EV fraction measured by RT-qPCR.
