# Supplementary material for: Extracellular vesicle‐encapsulated miR‐30c‐5p reduces aging‐related liver fibrosis
Source: Aging Cell. 2024 Sep 13;23(12):e14310. doi: 10.1111/acel.14310 (PMC11634720; doi:10.1111/acel.14310)
Supplement: Supplementary file 4 — Figure S4. [file ACEL-23-e14310-s008.pdf]

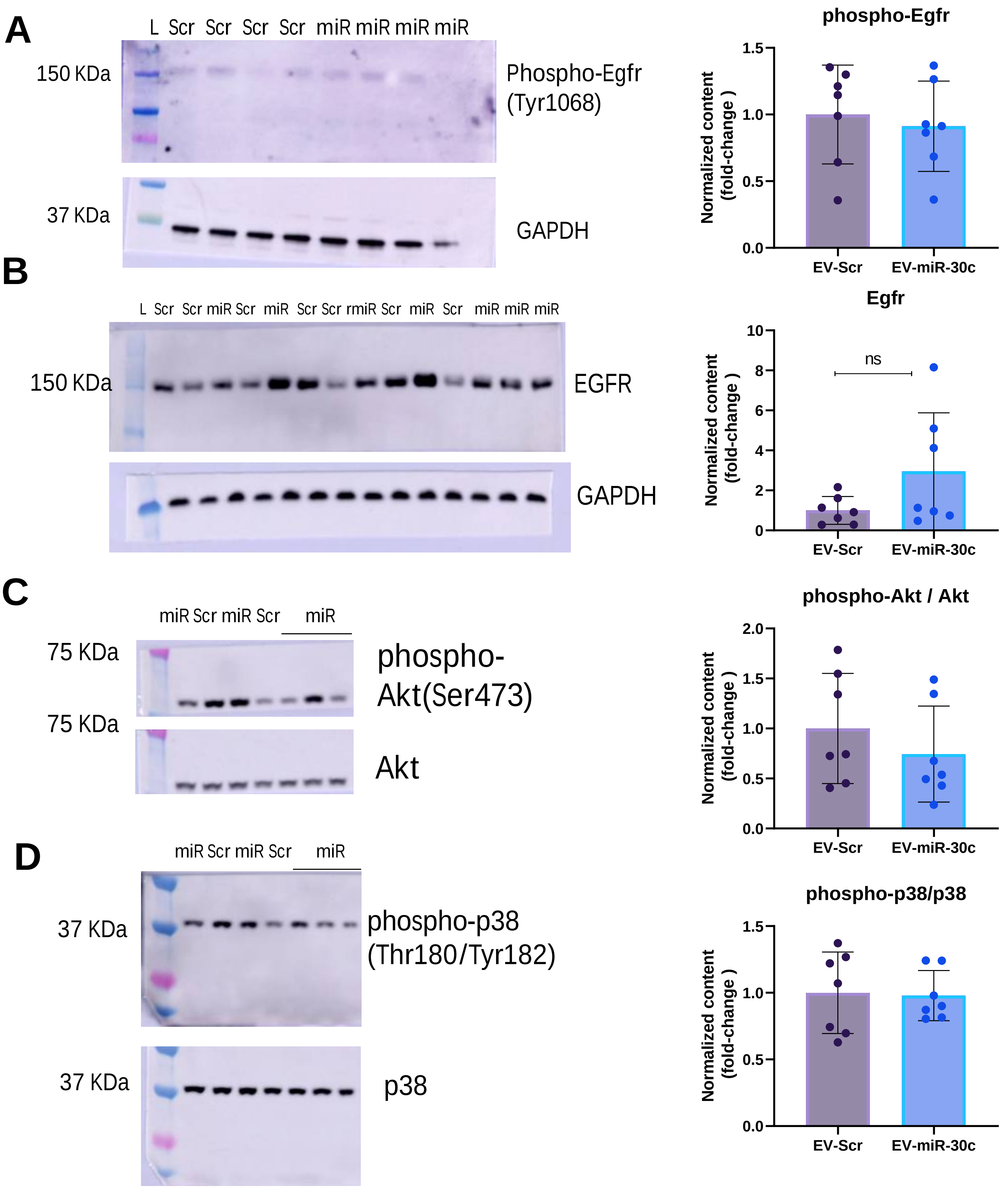
A
L Scr Scr Scr Scr miR miR miR miR
150 KDa
Phospho-Egfr (Tyr1068)
37 KDa
GAPDH
phospho-Egfr
Normalized content (fold-change)
EV-Scr
EV-miR-30c
B
L Scr Scr miR Scr miR Scr Scr rmiR Scr miR Scr miR miR miR
150 KDa
EGFR
GAPDH
Egfr
ns
C
miR Scr miR Scr miR
75 KDa
phospho-Akt(Ser473)
75 KDa
Akt
phospho-Akt / Akt
D
miR Scr miR Scr miR
37 KDa
phospho-p38 (Thr180/Tyr182)
37 KDa
p38
phospho-p38/p38

Figure S4: Therapy with extracellular vesicle-encapsulated miR-30c-5p (EV-miR-30c) does not affect Egfr or p38 signaling. Proteins from the liver from mice treated with EV-miR-30c or EV-Scr (negative control) were obtained and subjected to western blot to quantify protein levels. **A-D**: Left: Representative bands of western blot for Egfr and phospho-Egfr (Tyr1068), Akt and phospho-Akt (Ser473), and p38 and phospho-p38 (Thr180/Tyr182) and Gapdh; Right: Quantification of the optical density of the bands obtained in **A-D** using Image Studio. Levels of EGFR and phosphor-EGFR were normalized with Gapdh as they were stained in different membranes. Mir: EV-miR-30c-treated mice and Scr: EV-Scr-treated mice.
