# Supplementary material for: Extracellular vesicle‐encapsulated miR‐30c‐5p reduces aging‐related liver fibrosis
Source: Aging Cell. 2024 Sep 13;23(12):e14310. doi: 10.1111/acel.14310 (PMC11634720; doi:10.1111/acel.14310)
Supplement: Supplementary file 5 — Table S1. [file ACEL-23-e14310-s004.docx]

Table 1S: Primers´sequence used for quantification of mRNA by qPCR

| Offical Gene Symbol | Forward primer 5´- 3´ | Reverse primer 5´- 3´ |
| --- | --- | --- |
| *Acta2* | CAGGGAGTAATGGTTGGAAT | TCTCAAACATAATCTGGGTCA |
| *Angpt2* | AGAATAAGCAAGTCTCGCTTCC | TGAACCCTTTAGAGGCTCGGT |
| *Cdnk1* | ATATCCAGACATTCAGAGCCACA | CACTTTGCTCCTGTGCGGAA |
| *Cebpa* | TAGGTTTCTGGGCTTTGTGG | GATGGATCGATTGTGCTTCA |
| *Col1a1* | CGATGGATTCCCGTTCGAGT | GAGGCCTCGGTGGACATTAG |
| *Col3a1* | ACGTAAGCACTGGTGGACAG | CAGGAGGGCCATAGCTGAAC |
| *Col4a1* | GGCCCCAAAGGTGTTGATG | CAGGTAAGCCGTTAAATCCAGG |
| *Cs* | CTGCTCCAGTACTATGGCATGA | TTAAAGGCCCCTGAAACAAAACA |
| *Ccl2* | CACTCACCTGCTGCTACTCA | GCTTGGTGACAAAAACTACAGC |
| *Ccl6* | CACCAGTGGTGGGTGCATCAAG | GTGCTTAGGCACCTCTGAACTC |
| *Cdh1* | GGTCATCAGTGTGCTCACCTCT | GCTGTTGTGCTCAAGCCTTCAC |
| *Dgat1* | AGAAGAGGACGAGGTGCGA | GATGGCACCTCAGATCCCAGTAG |
| *Dll4* | GGGTCCAGTTATGCCTGCGAAT | TTCGGCTTGGACCTCTGTTCAG |
| *Eln* | CACCACCCCACCTCTTTGTG | CCAAAGAGCACACCAACAATCA |
| *F4/80* | TGACTCACCTTGTGGTCCTAA | CTTCCCAGAATCCAGTCTTTCC |
| *Fabp4* | TGAAATCACCGCAGACGACA | ACACATTCCACCACCAGCTT |
| *Fasn* | GATTCGGTGTATCCTGCTGTC | CATGCTTTAGCACCTGCTGT |
| *G6pc1* | GTCCAGGACCCACCAATACG | GGGCATCAATCTCCTCTGGG |
| *Gapdh* | CATCTTCCAGGAGCGAGACC | CTCGTGGTTCACACCCATCA |
| *Il1a* | ACGGCTGAGTTTCAGTGAGACC | CACTCTGGTAGGTGTAAGGTGC |
| *Il1b* | TGTGAAATGCCACCATTTGA | GGTCAAAGGTTTGGAAGCAG |
| *Il6* | TGATGCACTTGCAGAAAACA | ACCAGAGGAAATTTTCAATAGGC |
| *Irs1* | CCCTCTCAACAGCAGTCCCT | TTACGCTATTGACGATCCTC |
| *Irs2* | CACAATTCCAAGCGCCACAA | CATCACCTCCTCCCAGGGTA |
| *Mmp2* | AACGGTCGGGAATACAGCAG | GTAAACAAGGCTTCATGGGGG |
| *Mmp12* | TGGCCATTCCTTGGGGCTGC | GGGGGTTTCACTGGGGCTCCATA |
| *Pepck1* | GGCTCCGAGGAGGAGTACG | CAGTGAGAGCCAGCCAAAA |
| *Ppara* | TCGAATATGTGGGGACAAGG | TCTTGCAGCTCCGATCACAC |
| *Pparg* | CAAACCTGATGGCATTGTGAG | ATCTTAACTGCCGGATCCAC |
| *Ppia* | TATCTGCACTGCCAAGACTGAGT | CTTCTTGCTGGTCTTGCCATTCC |
| *Rpl19* | GAAATCGCCAATGCCAACTC | CTTCCCTATGCCCATATGCC |
| *Rpl0* | TAAAGACTGGAGACAAGGTG | GTGTACTCAGTCTCCACAGA |
| *Scd1* | CCACTCGCCTACACCAAC | GTGGTCGTGTAAGAACTGGAG |
| *Snai1* | TGTCTGCACGACCTGTGGAAAG | CTTCACATCCGAGTGGGTTTGG |
| *Srebp1c* | AAGCAAATCACTGAAGGACCTGG | AAAGACAAGGGGCTACTCTGGGAG |
| *Serpine1* | GCACAACCCGACAGAGACAA | ATGAAGGCGTCTCTTCCCAC |
| *Srfsf4* | TTCCCCGTACAATCTGGGC | GGTTCAGAACATAACAGACCGTTT |
| *Timp1* | GTAAGGCCTGTAGCTGTGCC | AGGTGGTCTCGTTGATTTCT |
| *Timp2* | TCAGAGCCAAAGCAGTGAGC | GCCGTGTAGATAAACTCGATGTC |
| *Timp3* | CCAGGATGCCTTCTGCAACT | AATAGTGTAGACCAGAGTGCCA |
| *Tgfr1* | TCTGCATTGCACTTATGCTGA | AAAGGGCGATCTAGTGATGGA |
| *Tnfa* | CCACCACGCTCTTCTGTCTAC | AGGGTCTGGGCCATAGAACT |
| *Tp53* | TCCGAAGACTGGATGACTGC | GATCGTCCATGCAGTGAGGT |
| *Vegfa* | GCACATAGAGAGAATGAGCTTCC | CTCCGCTCTGAACAAGGCT |
| *Vegfb* | GCCAGACAGGGTTGCCATAC | GGAGTGGGATGGATGATGTCAG |
| *Vim* | TCCAGAGAGAGGAAGCCGAA | AAGGTCAAGACGTGCCAGAG |
